# Multicompartment and cross-species monitoring of contaminants of emerging concern in an estuarine habitat^☆^

**DOI:** 10.1016/j.envpol.2020.116300

**Published:** 2021-02-01

**Authors:** Thomas H. Miller, Keng Tiong Ng, Aaron Lamphiere, Tom C. Cameron, Nicolas R. Bury, Leon P. Barron

**Affiliations:** a Department of Life Sciences, College of Health, Medicine and Life Sciences, Brunel University London, Kingston Lane, UB8 3PH, UK; b Department of Analytical, Environmental & Forensic Sciences, School of Population Health & Environmental Sciences, Faculty of Life Sciences and Medicine, King’s College London, 150 Stamford Street, London, SE1 9NH, UK; c School of Life Sciences, University of Essex, Wivenhoe Park, Colchester, Essex, CO43SQ, UK; d School of Science, Technology and Engineering, University of Suffolk, James Hehir Building, University Avenue, Ipswich, Suffolk, IP3 0FS, UK; e Suffolk Sustainability, University of Suffolk, Waterfront Building, Neptune Quay, Ipswich, IP4 1QJUK, UK; f Environmental Research Group, School of Public Health, Faculty of Medicine, Imperial College London, UK

**Keywords:** Occurrence, Pesticides, Pharmaceuticals, Environmental risk, Sediment, Invertebrate

## Abstract

The fate of many chemicals in the environment, particularly contaminants of emerging concern (CEC), have been characterised to a limited extent with a major focus on occurrence in water. This study presents the characterisation, distribution and fate of multiple chemicals including pharmaceuticals, recreational drugs and pesticides in surface water, sediment and fauna representing different food web endpoints in a typical UK estuary (River Colne, Essex, UK). A comparison of contaminant occurrence across different benthic macroinvertebrates was made at three sites and included two amphipods (*Gammarus pulex* & *Crangon crangon*), a polychaete worm (*Hediste diversicolor*) and a gastropod (*Peringia ulvae*). Overall, multiple contaminants were determined in all compartments and ranged from; <LOQ – 386 ng L^−1^ in surface water (n = 59 compounds), <LOQ – 146 ng g^−1^ in sediment (n = 39 compounds) and <LOQ – 91 ng g^−1^ biota (n = 33 compounds). *H. diversicolor* and *P. ulvae* (sediment dwellers) showed greater chemical body burden compared with the two swimming amphipod species sampled (up to 2.5 - 4-fold). The most frequently determined compounds in biota (100%, n = 36 samples) included; cocaine, benzyoylecgonine, carbamazepine, sertraline and diuron. Whilst some of the highest concentrations found were in species *H. diverscolor* and *P. ulvae* for psychoactive pharmaceuticals including citalopram (91 ng g^−1^), sertraline (69 ng g^−1^), haloperidol (66 ng g^−1^) and the neonicotinoid, imidacloprid (33 ng g^−1^) Sediment was noted as an important exposure route for these benthic dwelling organisms and will be critical to monitor in future studies. Overall, the analysis of multiple species and compartments demonstrates the importance of including a range of exposure pathways in order to appropriately assess chemical fates and associated risks in the aquatic environment.

## Highlights

• Multi-compartmental monitoring across three sites of an estuarine habitat is presented.
• The most impacted site was next to a WWTP discharge point.
• Emerging contaminants across four different macroinvertebrates reached up to 91 ng g^−1^.
• Infaunal species showed greater accumulation of contaminants than epibenthic organisms.
• A high proportion of contaminants determined in biota were psychoactive drugs.

## Graphical abstract

**Figure:**
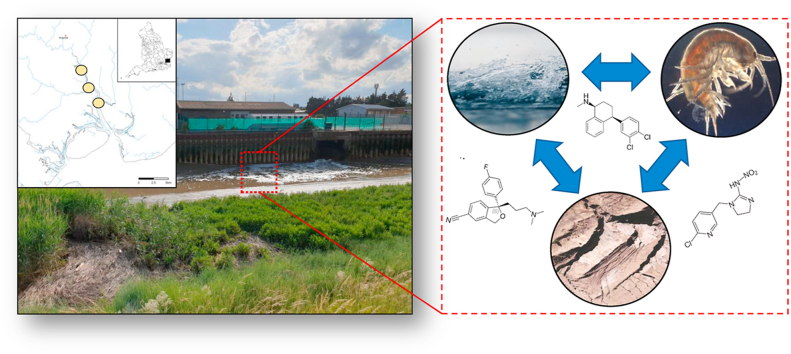

## Teaser

Multi-class contaminants of emerging concern were determined across surface water, sediment and macroinvertebrate species demonstrating widespread exposure in the aquatic environment.

## 1 Introduction

Anthropogenic activity is increasing pressure on both environmental and public health. One of these pressures is related to chemical contamination which has already led to some significant impacts in the environment (Desforges et al., 2018; Oaks et al., 2004). Many different classes of chemicals have now been found to occur in the environment that vary in terms of their persistence, bioaccumulation and toxicity. Two well-known classes of CEC that have been reported in the environment are pharmaceuticals and pesticides. Many studies have examined these two chemical classes for potential exposure and hazard in the environment, but there remain several knowledge gaps for reliable understanding of their potential risks (Naidu et al., 2016). Another class of chemicals often present in wastewater (that overlap with pharmaceuticals) are recreational drugs, but these have not had nearly as much focus as environmental contaminants by comparison, likely due to the obvious difficulties for regulation. Our previous study (Miller et al., 2019b) showed that several of these compounds were frequently found in surface water and biota which included cocaine, ketamine and 3,4-methylenedioxy methamphetamine (MDMA). These compounds overlap with sewage epidemiology studies that often report their occurrence in wastewater to link to public consumption (González-Mariño et al., 2020).

To understand risk and enable mitigation, it is critical to more fully understand exposure to different chemicals in the environment. This can be defined as the ‘exposome,’ a recent term that is extended from human toxicology into ecotoxicology and represents all potential exposures an organism will experience over its lifecycle (Escher et al., 2017). Surveillance of these chemicals exposures that make up the exposome can be used to prioritise chemicals of concern and/or regional areas of concern further directing mitigation and management strategies. However, within the aquatic environment, few studies have focussed on compartments beyond surface waters and fewer have determined chemicals across multiple compartments (Álvarez-Muñoz et al., 2015; Álvarez-Ruiz et al., 2015; Wang et al., 2014; Wilkinson et al., 2018), which is critical to gain a more holistic understanding of contaminant distribution and fate in the environment. Biomonitoring and measurement of internal concentrations have been recognised for many years as an important approach to understand chemical risk for biota (Connell, 2001; Connell et al., 1999; Sijm and Hermens, 2005). However, studies focussed on determination of internalised concentrations of chemicals have generally been limited (Escher et al., 2011; Miller et al., 2018). As a consequence, there have been large disparities reported in thresholds for exposure and hazard, which are based on extrapolated concentrations from exposure media. The bioavailability of chemical contaminants present in surface water and sediment will govern the uptake and accumulation by different organisms. By determining the internalised concentrations in biota, a better representative dose metric can link cause to effect and understand potential risk in the environment (Huerta et al., 2016; Margiotta-Casaluci et al., 2016; Munz et al., 2018). Thus, internal concentrations will be critical to refining current exposure thresholds that lead to toxicity.

The aim of this study was to characterise and better understand the exposure of CEC in an estuarine habitat through monitoring of multiple compartments. Pharmaceuticals, pesticides and recreational drugs were measured across multiple environmental compartments (sediment, water, biota) and multiple macroinvertebrate species at three sites along the estuarine River Colne (Essex, UK) downstream from the town of Colchester and its wastewater treatment works (e.g. Hythe). A previously validated analytical method for *Gammarus pulex* was applied to determine concentrations present across these different matrices and the selected species which included *G. pulex* (amphipod), *Peringia ulvae* (mollusc), *Hediste diversicolor* (polychaete, alternatively *Nereis diversicolor*) and *Crangon crangon* (amphipod). The characterisation of the chemical burden in multiple compartments and species will enable better understanding of chemical fate and subsequent risk to the aquatic environment.

## 2 Materials and methods

### 2.1 Reagents, chemicals and consumables

High performance liquid chromatography (HPLC) grade methanol, acetonitrile, and Liquid chromatography-mass spectrometry (LC-MS) grade (Optima™) ammonium acetate were purchased from Fisher Scientific (Loughborough, UK). A total of 141 compounds were targeted in this study. All analytical standards were of a purity of ≥97%. Ultra-pure water was obtained from a Millipore Milli-Q water purification system with a specific resistance of 18.2 MΩ cm or greater (Millipore, Bedford, MA, USA). Stock solutions (1 mg mL^−1^) were prepared in methanol (MeOH) or acetonitrile (MeCN) and stored in silanised amber vials (20 mL). Working solutions were prepared daily in ultra-pure water, as required. All solutions were stored at −20 °C and in the dark to reduce possible degradation.

### 2.2 Sample collection

**Fig. 1:**
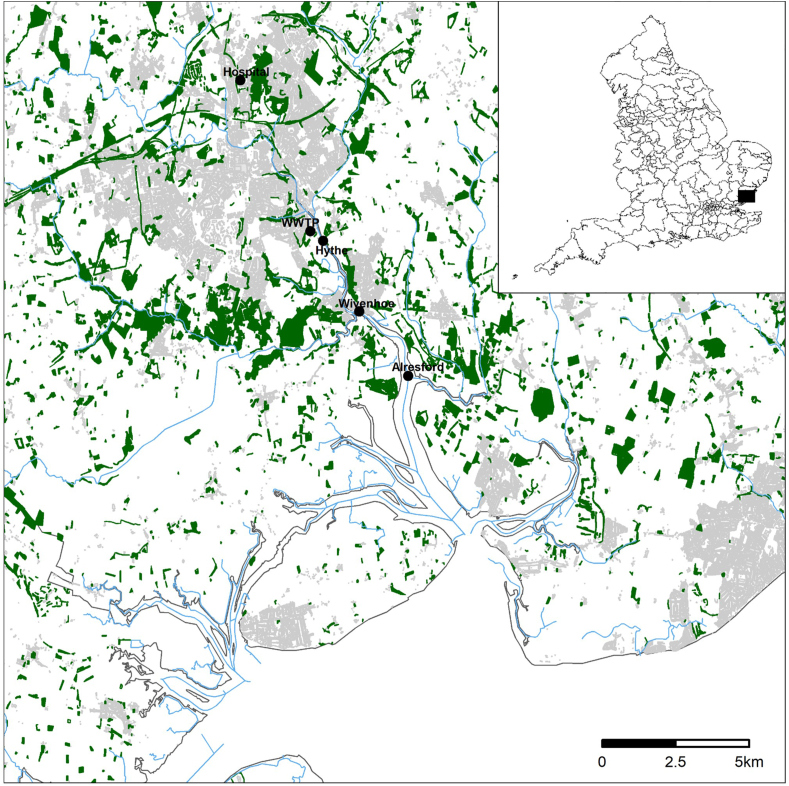
Location of sites for sample collection along the Colne Estuary. Hythe, Wivenhoe and Alresford were situated downstream of the major WWTP and are tidally influenced. Grey areas indicate buildings and green areas indicate woodland. Inset shows relative position to the rest of England. (For interpretation of the references to color in this figure legend, the reader is referred to the Web version of this article.)

Samples were collected in August 2019. Three locations were selected along the River Colne estuary (Essex, UK) which included Hythe, Wivenhoe and Alresford (Fig. 1). The first site Hythe was located downstream of a nearby wastewater treatment plant (WWTP) discharge point. All sites, Hythe, Wivenhoe (3.5 km) and Alresford (5.5 km), are tidally influenced as part of the Colne Estuary. All samples were taken on an ebb tide. Surface water samples (40 mL) were collected in Nalgene bottles in triplicate at each sampling site and transported in back to the lab in a cool box before subsequent storage at −20 °C.

Four species were selected for sampling including the sediment dwelling gastropod snail, *P. ulvae*, the sediment dwelling polychaete worm *H. diversicolor*, and two amphipods, *G. pulex*, and *C. crangon*. The amphipod *G. pulex* replaced *C. crangon* at the Hythe site due to lower salinity at this site. Amphipod species were collected by five kick samples into a 250 μm net per site. These were then sorted and transported back to the lab. Sediment dwelling species (*H. diversicolor* and *P. ulvae)* were collected from 20 cm mud cores in replicates of 4 per site. Animals were extracted through a 1 mm sieve and combined into species and sample specific replicate vials. Sediment/biota separation was completed within 3 h of sample collection to alleviate potential compound excretion from biota. All combined biomass macroinvertebrate replicates were stored at −20 °C prior to analysis. The remaining core sediment was separated into replicate specific vials (4 per site) and stored at 20 °C prior to analysis.

### 2.3 Sample preparation

The analytical workflow used for determination of compounds in animal, water and sediment samples followed a previous validated method for *G. pulex* (Miller et al., 2019b). Macroinvertebrate samples were lyophilised at −50 °C under vacuum for 24 h. Pooled samples of organisms were placed into 2 mL Eppendorf tubes with a 3 mm diameter tungsten carbide bead and subsequently ground into a fine powder using a TissueLyser LT (Qiagen, Hilden, Germany) set at 50 Hz for 5 min. Freeze-dried composite samples homogenised material (20 mg) was transferred to a new 2 mL Eppendorf with any necessary spiking carried out directly onto the solid matrix using a 100 μL volume of an appropriate working solution for matrix matched calibration curves.

A 2 mL volume of 3:1 (MeCN:H_2_O) acidified with 0.1% (v/v) glacial acetic acid was added to the material and agitated for 5 min at 50 Hz in the Tissuelyser LT (Qiagen, Hilden, Germany). The samples were then placed in an ultrasonic bath for 15 min followed by centrifugation for 5 min at 14,000 rpm to pellet insoluble particulate matter. Following extraction and settling, an aliquot of the supernatant (1.9 mL) was diluted to 100 mL with 10 mM ammonium acetate in ultra-pure water (pH 6.5). Tandem solid phase extraction (SPE) was then carried out on the diluted sample using a Strata Alumina-N cartridge (6 mL, 1 g, Phenomenex Ltd., Cheshire, UK) coupled to an Oasis HLB cartridge (6 mL, 200 mg, Waters Corp., Hertfordshire, UK). Before loading of the sample, the combined SPE cartridges were first conditioned with 6 mL of methanol and 6 mL of ultra-pure water with 10 mM ammonium acetate. After sample loading, both cartridges were then washed with 1 mL ultra-pure water and dried for ∼30 min under vacuum. The alumina cartridge was then discarded and the HLB cartridges were stored at −20 °C until analysis. Cartridges were eluted with 5 mL MeOH (2 × 2.5 mL volumes) and dried under pure nitrogen (99.9%, 1.0 bar) at 35 °C using a TurboVap LV (Biotage, Uppsala, Sweden).

For sediment, samples were lyophilised as above and then 50 mg of material was weighed and placed in a 2 mL Eppendorf tube with a tungsten carbide bead (3 mm). The extraction and clean-up method were the same as described above but without the use of Strata Alumina-N cartridges.

For surface water, samples (20 mL) were filtered through a 0.45 μm glass-fibre filter and were subsequently cleaned-up by the same SPE method described above without the use of the Strata Alumina-N cartridges.

All extracts (animal, sediment and water) were reconstituted in 0.1 mL 90:10 (v/v) 10 mM ammonium acetate in H_2_O:MeCN transferred to 200 μL silanised glass inserts, held within a 2 mL amber autosampler vial. After reconstitution, samples were immediately analysed using liquid chromatography tandem mass spectrometry (LC-MS/MS) method described below.

### 2.4 Instrumental analysis and conditions

Analytical separations were performed on a Nexera X2 LC system (Shimadzu, Kyoto, Japan) using a Raptor™ biphenyl column (100 × 2.1 mm, 2.7 μm particle size) (Thames Restek, Saunderton, UK) and a Raptor™ biphenyl guard column (5.0 × 2.1 mm, 2.7 mm particle size) (Thames Restek, Saunderton, UK), which was housed within an EXP® Direct Connect Holder (Thames Restek, Saunderton, UK). An injection volume of 20 μL with 0.3 mL min^−1^ flow rate was used. Mobile phases were 90:10 (v/v) 10 mM ammonium acetate in H_2_O:MeCN (A) and 20:80 (v/v) 10 mM ammonium acetate in H_2_O:MeCN (B). The gradient elution profile followed a linear ramp of mobile phase B which increased to 10% at 1 min, 35% at 5.6 min, 40% at 7 min, 50% at 8 min and 100% at 11 min and was held for a further 11 min before returning to initial conditions. Re-equilibration time was 3 min resulting in an overall run time of 25 min. Detection and quantification were performed using an 8060 triple quadrupole mass spectrometer with electrospray ionisation (ESI) interface (Shimadzu, Kyoto, UK). Pureshield argon was used as the collision-induced dissociation gas (BOC Gases, Guildford, UK). Nitrogen and dry air were generated using Genius 1051 gas generator (Peak Scientific, Inchinnan, UK). Mass spectrometry (MS) was performed in multiple reaction monitoring (MRM) mode using positive–negative ionisation polarity switching. MRM optimisation of each precursor was performed using the LabSolutions optimisation for method (version 5.93, Shimadzu, Kyoto, Japan), where individual solutions for each analyte in methanol at 1.0 μg mL^−1^ was injected (10 μL) at 0.5 mL min^−1^ to an isocratic profile; 30% mobile phase A and 70% mobile phase B. One MRM event was acquired for subsequent quantification with a second transition for identification, where possible. Chromatographic data was acquired by LabSolutions (version 5.93, Shimadzu, Kyoto, Japan) and processed using LabSolutions Insight (version 3.2, Shimadzu, Kyoto, Japan). See the SI for full details of analytical conditions (Table S1 & 2).

Whilst the analytical method was validated for *G. pulex*, it was applied to the other matrices where in-depth method performance assessment data is not available. Nevertheless, quantification was performed using matrix-matched calibration for each matrix type. For animal samples, a total of four calibration curves were prepared at 0.5, 1, 5, 10, 25 and 50 ng g^−1^ for each individual species to be analysed (the same species were pooled across sites for the calibration). Sediment calibration curves were prepared for each site at 0.5, 1, 5, 20, 50 and 100 ng g^−1^ leading to a total of three separate calibration curves. Additionally, compounds quantified in sediment samples were assessed for method repeatability at two concentrations of 20 ng g^−1^ and 100 ng g^−1^ (see Table S3). The surface water calibration curve was prepared by pooling individual sites (20.0 mL per site) into a composite matrix and spiking at 5, 20, and 80 ng L^−1^. Pre-extraction spikes were added using 100 μL of an appropriate working solution containing the full mixture of analytes (stored in MeCN). Neat samples (i.e. containing no spikes) were run in triplicate to background correct when performing quantifications. Calibration curves, where necessary, were normalised against stable isotopically labelled internal standards (SIL-IS) that were spiked at a constant concentration (50 ng g^−1^ for solid samples, 100 ng L^−1^ for surface water) between calibration points and in the environmental samples (see SI for details). Quantifications were only performed where linearity was acceptable (R^2^ ≥ 0.98). Analytes were reported below the limit of detection or quantification when the corresponding peak was below a signal-to-noise threshold of 3:1 to 10:1, respectively.

## 3 Results and discussion

### 3.1 Contamination across different macroinvertebrate species

Four species of macroinvertebrate representing three potential food web routes were sampled in this study including amphipods (e.g. *G. pulex* or *C. crangon*), a gastropod (*P. ulvae*) and a polychaete (*H. diversicolor*). The macroinvertebrate species sampled occupy different ecological niches. In order to understand the implication of CEC, it is important to understand whether exposure in these organisms vary and what role they have in estuarine food-webs. For example *P. ulvae* is a sediment dwelling biofilm grazer and is a large dietary component of estuarine birds (Burton, 1974; Patterson, 1982). *H. diversicolor* is a predatory polychaete and is a large dietary component of estuarine birds and fish (Burton, 1974; Green et al., 2009). A comparison between average total body burden in each species for the three sites showed that *H. diversicolor* had higher average concentrations of contaminants measured at both Hythe (8.0 ng g^−1^) and Wivenhoe (4.2 ng g^−1^). In contrast, *P. ulvae* showed a higher average body burden of 4.1 ng g^−1^ at Alresford but contaminants were more evenly distributed when compared to *H. diversicolor* (3.1 ng g^−1^) and *C. crangon* (3.0 ng g^−1^). The reduction in average body burden for these species at Wivenhoe and Alresford as mentioned above is due to the sites being located further downstream from the discharge point of the WWTP. Overall, the data shows that *H. diversicolor* had higher accumulation of the targeted chemical contaminants. Studies in the lab have shown that species life stage and traits can affect uptake and elimination processes (Rubach et al., 2010a; 2010b). This species burrows into sediments, is a generalist including predatory behaviours and might represent an important exposure route as this compartment was also shown to have highest average burden across all compartments measured in this study.

Amphipods have been a commonly used organism for biomonitoring studies with many authors using gammarids to determine concentrations of CEC (Miller et al., 2015; Inostroza et al., 2016; Sordet et al., 2016; Munz et al., 2018; (Miller et al., 2019b)). These are generally seen as an ecologically important species for their role in nutrient cycling. However, in this study this *Gammarus* showed the lowest body burden at Hythe and was also the case for *C. crangon* at Wivenhoe and Alresford. The lower concentrations determined in the amphipods suggest that these organisms might be a more conservative bioindicator to assess exposure in the environment. However, few studies have looked at chemical contamination across different macroinvertebrate species in the field. An investigation into occurrence of estrogenic compounds in Taihu Lake, China compared concentrations of E1, E2, E3, EE2 and BPA in a fish, clam and snail species (Wang et al., 2014). Concentrations detected in the species were dependent on the site, but high concentrations determined in the sediment led to largest concentrations observed in the snails reaching up to ∼1 mg kg^−1^. This reiterates the observation here, that sediment can be an important exposure route for benthic dwelling organisms and is not often investigated in occurrence studies. In marine bivalves collected from the Ebro Delta in Spain, the oyster *Crassostrea gigas* was shown to have higher measured concentrations of several pharmaceuticals when compared to two mussel species including *Mytilus* spp., and *Chamalea gallina* (Álvarez-Muñoz et al., 2015). A third study (Wilkinson et al., 2018) that investigated multiple contaminant classes in two benthic invertebrates (*G. pulex* and *Bithynia tentaculata*) showed similar measured concentrations for pharmaceuticals and recreational drugs but varied more widely for plasticisers and perfluorinated compounds, with *B. tentaculata* accumulating up ∼10-fold more than *G. pulex*. This previous study also estimated sediment bioaccumulation factors (BSAF) which indicated that sediment was a more significant exposure route than surface water.

### 3.2 Pharmaceutical exposure

Pharmaceuticals detected at high concentrations included haloperidol, sertraline and imidacloprid. Haloperidol was detected at higher concentrations in *H. diversicolor* at both Hythe and Wivenhoe with mean concentrations of 35.5 ng g^−1^ and 23.3 ng g^−1^, respectively. However, haloperidol and sertraline were again determined at lower concentrations in *G. pulex* than when compared with *P. ulvae* and *H. diversicolor*. Haloperidol was determined in *G. pulex* in our previous study (Miller et al., 2019) but was only measured once at 5.3 ng g^−1^ in a comparatively rural catchment. *G. pulex* showed low measured concentrations of haloperidol in the present study and was also low in *C. crangon*. This compound was determined to have a high likelihood of an effect occurring in the environment due to the low human therapeutic plasma concentration to elicit a pharmacological effect (1 ng mL^−1^) (Fick et al., 2010). However, this compound has not been previously reported in the literature and further investigation into potential effects would be necessary.

Over half of the pharmaceuticals detected in biota samples were psychoactive drugs including carbamazepine, amitriptyline, memantine, diazepam, citalopram, nordiazepam, venlafaxine, clozapine, temazepam, sertraline, haloperidol and risperidone. These types of contaminants have recently been gaining attention for their potential for sub-lethal effects on behaviour which current risk assessments do not account for (Bláha et al., 2019; Brodin et al., 2013; Huerta et al., 2016). These compounds often have low therapeutic doses and typically designed to be more hydrophobic to permeate the blood brain barrier (Iyer et al., 2002; Tanoue et al., 2019). Thus, accumulation and the potential for effects might be increased for these compounds in the environment. Several authors have shown various effects for SSRI compounds including sertraline, citalopram, fluoxetine and benzodiazepines such as diazepam, oxazepam and temazepam (Bossus et al., 2014; Martin et al., 2017; Valenti et al., 2012). It has been demonstrated that non-target exposure to many of these compounds can produce inimical effects in the form anxiolytical, physiological and behavioural responses. For example, environmentally relevant exposure to SSRI’s has been found to reduce locomotor activity and feeding efforts in the three-spine stickleback (Kellner et al., 2018), decrease body size in juvenile brown trout (Ziegler et al., 2020), and promote premature larval release in freshwater mussels (Hazelton et al., 2013). These compounds have also been found to elicit adverse effects at a population level in vertebrate species including effects on sexual selection in the male Mosquitofish *Gambuisi holbrooki* (Bertram et al., 2018), and the modification of courtship behaviour in the male Starling *Sturnus vulgaris* (Whitlock et al., 2018).

**Fig. 2:**
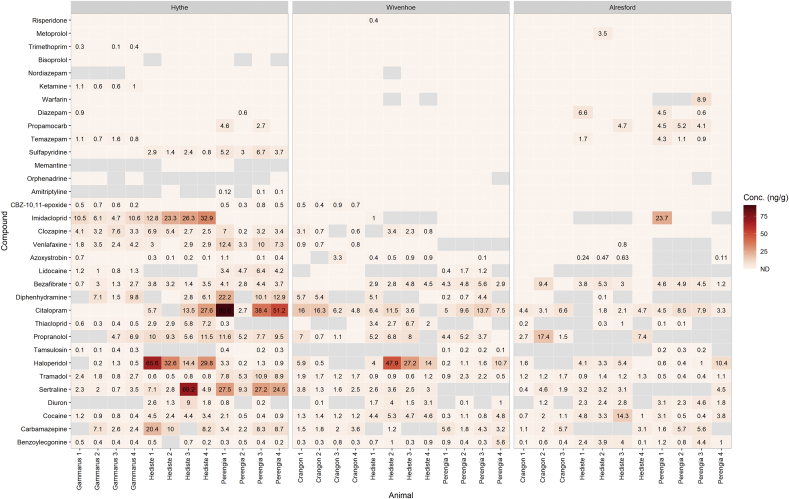
Heatmap showing concentrations (ng g^−1^) of CEC in the four macroinvertebrate species collected from each sampling site. Grey tiles indicate samples were below the limit of quantification.

The highest measured concentration of single contaminant in the macroinvertebrates reached 90.8 ng g^−1^ for the SSRI citalopram in *P. ulvae* at Hythe followed by sertraline (69.2 ng g^−1^) and haloperidol (65.6 ng g^−1^) in *H. diversicolor* (Fig. 2). The compound citalopram was also determined at high concentrations in *H. diversicolor* and while not detected in *G. pulex,* it was in *C. crangon*. *P. ulvae* can make up 89.5% of the diet of estuarine birds, which also consume sediment while foraging upon them. Shelduck have been found with up to 3000 individuals ingested (Anders et al., 2009), whilst acknowledging that dietary transfer has not been demonstrated here, there is a potential risk to foraging vertebrates in estuaries that are most often both internationally protected for shorebirds and universally exposed to WWTP effluent containing biologically active behavioural modifying drugs. The focus of these behavioural compounds in the literature likely stems from their high consumption, but future studies should aim to measure exposure to other psychoactive drugs to ensure non-bias of targeted analyte lists (i.e. the Matthews Effect (Daughton, 2014)). As such, exposomics is a developing field that is benefitting from established workflows developed for metabolomic studies that focus on untargeted analysis. By utilising these approaches, characterisation of exposure in the environment will be improved.

### 3.3 Pesticide exposure

The neonicotinoid pesticide imidacloprid was not detected in *P. ulvae* but measured on average 24 ± 8 ng g^−1^ in *H. diversicolor* and 8 ± 3 ng g^−1^ in *G. pulex*. This is similar to concentrations that have been previously reported in the literature that measured up to 21 ng g^−1^ in *G. pulex* (Munz et al., 2018). However, very few other studies have measured neonicotinoid presence in biota and so more general trends are difficult to establish. Only one other neonicotinoid was detected in biota and was present at relatively lower concentrations (thiacloprid), that was <1 ng g^−1^ in *G. pulex* and ranged from 2 to 7 ng g^−1^ in *H. diversicolor*. Three neonicotinoids were subject to regulations published May 2018 (European Commission, 2016a; 2016b) (with a grace period up to December 2018) by the European Commission which banned all outdoor uses and seed treatments of imidacloprid, clothianidin and thiamethoxam, with the exception of greenhouse use (European Commission). Thiacloprid while not subject to these regulations, is a potential endocrine disruptor and has been recommended as a candidate for substitution (European Commission). Acetamiprid was evaluated by EFSA to present a low hazard to pollinators and no further restrictions were applied (European Food Standards Agency, 2016). Pesticide usage in the UK is estimated by the Department of Environment, Food and Rural Affairs (Defra)(Department of environment food and rural affairs, 2020). Interestingly, 2018 surveys revealed that compared to 2016 levels, acetamiprid use for arable crop growth decreased by 59%, whereas thiacloprid usage has increased by 58%. Additionally, no usage of imidacloprid has been reported in these surveys. In terms of measured occurrence, it is possible that persistence of these compounds in soils could lead to continued exposure in waters through leaching. Imidacloprid and clothianidin have been shown to have the longest soil half-lives of 191 and 545 days, respectively (University of Hertfordshire, 2007). However, clothianidin whilst more persistent was not detected in any of the compartments sampled and was used in larger quantities than imidacloprid (79.2 tonnes compared with 0.2 tonnes in 2016). Thus, it difficult to pinpoint the source of imidacloprid in the aquatic environment. However, neonicotinoids are also used in veterinary medicine (e.g. tick/flea control) and might represent an additional route of exposure for these substances to enter the aquatic environment beyond agricultural use. Other detected pesticides included propamocarb, azoxystrobin and diuron. These three pesticides are all approved under current European Commission regulations. However, whilst azoxystrobin and propamocarb are currently used in the UK, diuron usage has not been reported since 2016 (0.2 tonnes) (Department of environment food and rural affairs, 2020). The detection in biota might be related to the persistence in sediment as it was detected in this compartment but not in surface water.

### 3.4 Recreational drug exposure

Cocaine and its metabolite benzoylecgonine were detected in every animal, sediment and surface water sample, demonstrating high frequency occurrence that was also reported in our previous study for rural Suffolk for both biota and surface water (Miller et al., 2019b). Surprisingly, average concentrations for cocaine in this study were 2.5 ± 2.6 ng g^−1^, in contrast to the average concentrations determined in Suffolk (5.9 ± 4.3 ng g^−1^). Land use near the sampling sites in Suffolk were much less urbanised areas with considerably smaller populations thus contamination was expected to be higher in the present study. Cocaine is the second most used recreational drug in the UK (below cannabis) (Home Office, 2019). Ketamine was only detected in *G. pulex* at Hythe at low concentrations (≤1.1 ng g^−1^). This compound however was detected in all surface water and sediment samples. Whilst this compound is used in veterinary medicine, its misuse has increased with larger rises recorded between 2016 and 2018 (Home Office, 2019). Other potential recreational drugs included tramadol, diazepam and temazepam but these also have medical uses. Tramadol was determined at higher concentrations in *P. ulvae* at both Hythe and Wivenhoe when compared with the other macroinvertebrate species.

Very few studies exist that have looked at the potential effects of recreational drugs in animals outside captivity and as with pharmaceutical pollution this requires further consideration for potential environmental risk. Regulation on use may not be possible with recreational drugs and so other innovative solutions would be needed. For example, schemes that aim to treat drug abuse as a public health issue rather than using traditional law and order approaches (Mold, 2018; Volkow et al., 2017) could have additional benefits in this scenario by reducing the number of users and subsequently reducing input into the environment.

The number of contaminants determined across four species of macroinvertebrates representing three unique food web routes is concerning, but without better understanding of potential for effects it is beyond the scope of this study to link to risk. Nevertheless, this study does demonstrate that there are cross-species differences in exposure which might lead to some species being more susceptible to effects of environmental contaminants. As a final consideration we should focus on characterising exposure in biota as this represents the at-risk group from the potential hazards of chemical contaminants in the environment. Particularly, as it is challenging to link exposure between different compartments without further mechanistic studies on bioavailability and accumulation.

### 3.5 Characterising contamination of sediment and surface water

The measurement of contaminants in abiotic compartments has been prioritised in previous monitoring studies with surface waters often accounting for most measurements. For example, with pharmaceuticals, only 2% of measured data (up to October 2013) was determined in sediment compared with surface waters which accounted for 55% of data (total of 123, 761 measured datapoints)(aus der Beek et al., 2016). Regarding, recreational drugs there have been a limited number of studies focusing on occurrence beyond wastewater and surface water. Very few researchers have characterised recreational drugs in biota (Klosterhaus et al., 2013; Wilkinson et al., 2018; (Miller et al., 2019b)) and sediment (Álvarez-Ruiz et al., 2015; Klosterhaus et al., 2013; Langford et al., 2011; Wilkinson et al., 2018).

### 3.6 Sediment

**Fig. 3:**
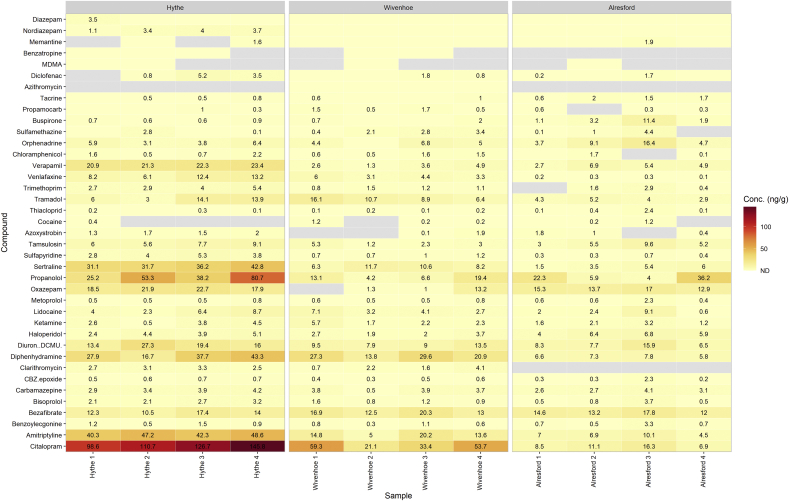
Heatmap showing concentrations (ng g^−1^) of CEC determined in the sediment cores collected from each sampling site. Grey tiles indicate samples were below the limit of quantification.

Sediment acts as an additional route of exposure for benthic-dwelling organisms and potential re-mobilisation of adsorbed chemical contaminants (especially at periods of high flow or tidally influenced rivers). The highest measured concentration was for the compound citalopram at Hythe that reached up to 145.8 ng g^−1^ (mean: 120.5 ng g^−1^) (Fig. 3). Other compounds that reached higher concentrations at Hythe included propranolol (mean: 49.4 ng g^−1^), amitriptyline (mean: 44.6 ng g^−1^), sertraline (mean: 35.5 ng g^−1^), diphenhydramine (mean: 31.4 ng g^−1^), verapamil (mean: 22.0 ng g^−1^), oxazepam (mean: 20.3 ng g^−1^), diuron (mean: 19.0 ng g^−1^) and bezafibrate (mean: 13.6 ng g^−1^). These compounds were also present at higher concentrations at Wivenhoe and Alresford in comparison to the remaining contaminants detected. The sediment at all three sites were broadly similar estuarine muds (silt & clay) with 99% of particles <1 mm by mass. Sorption of chemical contaminants is via several different mechanisms including cation exchange, hydrophobic interaction, hydrogen bonding and surface complexation (Tolls, 2001). The higher occurrence of propranolol might be related to its high partition coefficient (*K*_d_) which was shown to be the largest compared with several other beta-blockers for two different sediment types (Ramil et al., 2010). In addition to non-polar interactions, propranolol will likely interact via hydrogen bonding between hydroxyl groups with free silanol in sediment. Previous studies have noted that pH and sediment type/composition can have significant influence on the dominant mechanism of sorption (Jones et al., 2006; Schaffer et al., 2012). Furthermore, hydrophobicity is unreliable for prediction of the fate of pharmaceuticals, which has also been noted for uptake in biota (Chang et al., 2019; Miller et al., 2019a; Schaffer et al., 2012). A potentially important route for sorption of pharmaceuticals is cation exchange as sediment surfaces often have an associated negative charge (Martínez-Hernández et al., 2014). A total of three pesticides were determined in the sediment samples with the remaining compounds being pharmaceuticals and recreational drugs. Whilst most drugs are either basic or acidic, a higher proportion are basic (57% basic, 29% acidic from a 582 compound dataset) and this trend is more pronounced for CNS drugs where the distribution of basic compounds increased to 75% (Manallack, 2007). The higher proportion of basic drugs for CNS treatments is related to penetration of the blood brain barrier (BBB) where functional amines can favour transport across this membrane. This may further suggest that sediments are an important compartment regarding drug transport and fate in freshwater systems, particularly for psychoactive drugs.

### 3.7 Surface water

Surface water was the most contaminated compartment in terms of number of unique compounds determined, but the data further demonstrate that occurrence in water does not translate well into concentrations present in biota. For example, of the 70 unique compounds determined across all three aquatic compartments, 24 compounds were determined in surface water only and a further 9 compounds were determined in both surface water and sediment, but not present in biota (Fig S1). Another 7 compounds were not detected in surface water but were present in biota only or both biota and sediment. The issue when compared to sediment and biota samples, is that surface water samples normally represent a single “snapshot” in time, unless using passive samplers or high frequency composite samplers. Multiple compounds measured in the surface water of this current study showed large variations in measured concentrations and is likely due to the high spatiotemporal variability associated with surface water.

**Fig. 4:**
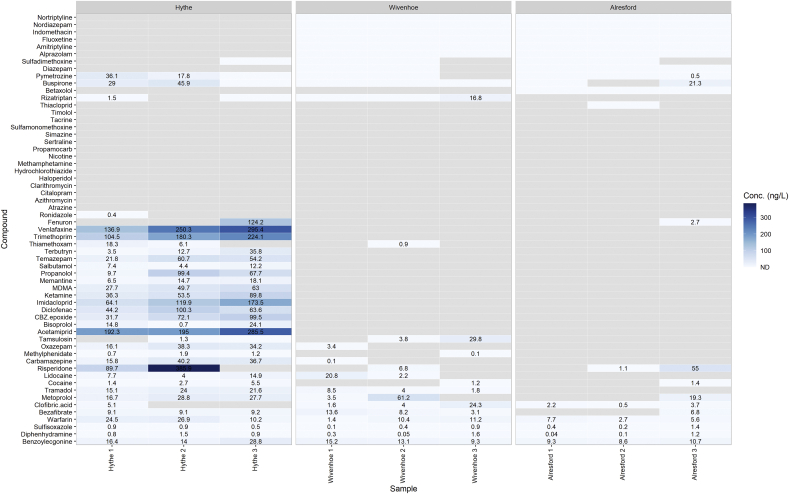
Heatmap showing concentrations (ng L^−1^) of CEC determined in surface water collected from each sampling site. Grey tiles indicate samples were below the limit of quantification.

The highest concentration determined in surface water was 386 ng L^−1^ corresponding to risperidone which is an antipsychotic medication (Fig. 4). Additional compounds detected at relatively higher concentrations included venlafaxine (antidepressant), acetamiprid (neonicotinoid), imidacloprid (neonicotinoid) and trimethoprim (antibiotic). Of the compounds determined at Hythe many of these compounds were reduced below the LOQ by the second site Wivenhoe. Other studies have shown similar trends where concentrations further downstream of WWTPs are significantly reduced (Baker and Kasprzyk-Hordern, 2013; Munro et al., 2019). Cocaine and its metabolite BZE were also detected at a mean value of 3.2 ng L^−1^ and 19.7 ng L^−1^, respectively. The ratio of cocaine:BZE is 0.16 which is similar to ratios found in other surface waters (Munro et al., 2019). This ratio indicates that the input into the river is likely to stem from untreated sewage (influent) (Baker et al., 2014) entering the River Colne from combined sewer overflows. In 2019, the Colchester storm overflow upstream of the Hythe sampling point, spilled 342 times totalling 7248 h (302 days) of untreated wasted entering the River Colne (The Rivers Trust). Other recreational drugs determined at higher concentrations included ketamine and MDMA and two benzodiazepines (temazepam and oxazepam). Diazepam was not quantifiable, but this compound is extensively metabolised (>90%) to temazepam and oxazepam. Therefore, measured levels of these two compounds will also be related to diazepam consumption.

Of the pharmaceuticals detected, cardiovascular drugs were frequently detected across all sites and reached higher concentrations at Hythe which included the anticoagulant warfarin (11 ng L^−1^) and the beta-blockers; propranolol (59 ng L^−1^), metoprolol (26 ng L^−1^), bisoprolol (13 ng L^−1^) and timolol (<LOQ). Carbamazepine and its metabolite carbazmepine-10,11-epoxide were both detected reaching an average of 31 ng L^−1^ and 68 ng L^−1^ at Hythe, respectively. This drug is extensively metabolised, with the major route to the epoxide form (and is the pharmaceutically active species being a pro-drug). However, the drug is considered persistent due to it limited removal during wastewater treatment and presence across multiple environmental compartments (Zhang et al., 2008).

Four of the seven neonicotinoids available on the market were detected here. Acetamiprid and imidacloprid averaged 181.5 ng L^−1^ and 119.2 ng L^−1^, respectively, whereas thiamethoxam measured 15.3 ng L^−1^ and thiacloprid was only detected below LOQ. However, all neonicotinoids were measured below the LOQ at Wivenhoe and Alresford. This suggests that the source of input may come from the discharge point of the WWTP as opposed to run-off or leaching. As mentioned previously, it is difficult to identify the source of neonicotinoid presence in this study, but it is a possibility that the occurrence is related to wastewater from indoor agricultural practices or potentially from veterinary use. Wastewater from greenhouses have been recognised as a contributor to pollution related to high nutrient content such as phosphate (Dunets and Zheng, 2014; European Commission, 2015) and thus could be have the potential to be further linked as a source for micropollutants. Further investigation is warranted into this aspect as few studies have looked at indoor agriculture practices regarding pollution and therefore regulations surrounding indoor pesticides may need revision to be protective of the environment.

To summarise, increasing our surveillance of the aquatic environment by monitoring multiple compartments and species will increase our characterisation of the exposome, whilst overcoming limitations with different sampling approaches, further improving our understanding of environmental exposures.

### 3.8 Occurrence of pharmaceuticals, recreational drugs and pesticides across multiple compartments

Research efforts for the determination of pharmaceuticals and pesticides in the environment have primarily been placed on the measurement of these chemicals in abiotic compartments such as surface waters, ground waters and marine waters (aus der Beek et al., 2016). A holistic understanding of exposure requires consideration of live biota exposed to these abiotic compartments. Consideration of multiple species is relevant as species that occupy different ecological niches and roles within food-webs are likely to receive and transport contaminants in unique ways. The compounds detected in this study included pharmaceuticals, pesticides and recreational drugs, with pharmaceuticals being the most frequently determined compounds and present at higher concentrations relative to the detected pesticides and recreational drugs.

Overall and out of 141 compounds included in the analytical method; a total of 33 compounds were detected in the macroinvertebrates sampled, 39 compounds detected in sediment samples and 59 compounds detected in surface water samples. The most contaminated site was Hythe (Fig. 1), closely located downstream of a WWTP and accounts for both the elevated concentrations and diversity of detected chemicals in comparison to Wivenhoe and Alresford. All three sites are tidally influenced and have periods of ebb and flow, where the tide moves inland (flow) and then drains outward (ebb). A previous investigation into the River Thames (Munro et al., 2019) showed that tidal cycles led to homogeneity across a majority of quantified pharmaceuticals and recreational drugs due to mixing from multiple combined sewer overflow and treated effluent influx points in the Central London region. The WWTP located upstream of the Hythe sampling site, serves a population equivalent of 131,413 with primary, secondary and UV treatment stages in place (Office International de l’Eau).

**Fig. 5:**
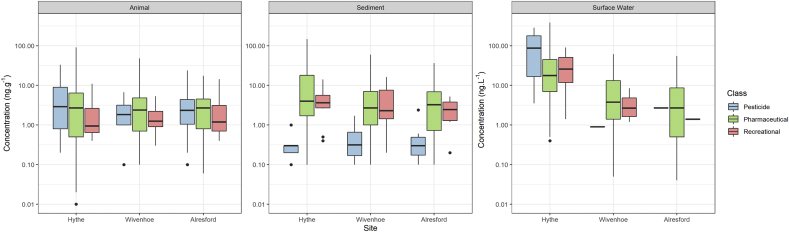
The concentration ranges of chemical contaminants determined across each of the three sites and the three compartments (e.g. surface water, sediment and biota). Concentrations for solid samples are based on dry weight.

Concentrations of the chemical contaminants generally decreased further downstream from the WWTP for all sample types, but this was most apparent for surface water samples (Fig. 5). Previous studies have also shown that WWTP discharges are a significant source of contamination and for sites located inland, concentrations are generally lower due to dilution by coastal waters (Biel-Maeso et al., 2018; Čelić et al., 2019). Decreases in concentrations in sediment and biota samples were less apparent between sites particularly for Wivenhoe and Alresford as the ebb and flow of the tide will affect the mixing of sediment and surface water between the two sites.

The majority of compounds determined were pharmaceuticals with fewer pesticides and recreational drugs detected. For example, in the biota samples 33 compounds were detected; 25 were pharmaceuticals (76%), 4 pesticides (diuron, propamocarb, thiacloprid & imidacloprid), 2 recreational drugs (cocaine & ketamine) and 2 metabolites (benzoylecgonine & carbamazepine-10,11-epoxide). Within the pharmaceutical class, 13 compounds (52%) were psychoactive drugs including antidepressants and antipsychotics. There are a range of potential sources of behavioural drugs in this urban WWTW catchment, from well documented increases of use in the general population to a main regional hospital. In the UK, most direct emissions from hospitals into sewerage is prohibited (Water UK, 2014) and so occurrence in the environment is most likely to come from domestic wastewater after human consumption which may explain the relatively low contributions of hospital effluent to WWTP influent contaminant loads in previous works (Verlicchi et al., 2012).

**Fig. 6:**
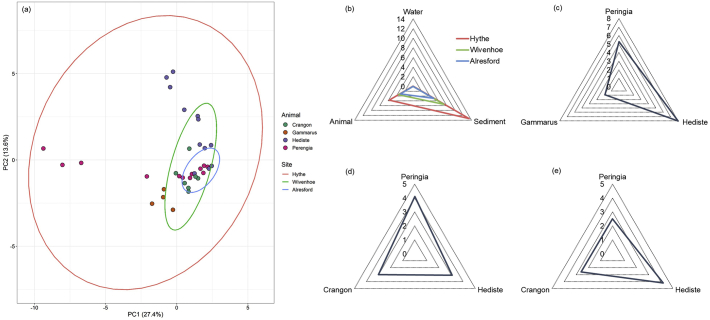
Comparison of chemical burden across sampling sites, compartments and species. **(a)** Principal component analysis showing the variance in the chemical burden in biota between sites with ellipses representing the 95% confidence interval. **(b)** Mean chemical burden for each compartment sampled in the Colne Estuary, **(c)** mean chemical burden determined in macroinvertebrates collected from Hythe, **(d)** mean chemical burden determined in macroinvertebrates collected from Wivenhoe and **(e)** mean chemical burden determined in macroinvertebrates from Alresford. All radar plots are based on a part per billion (ppb) scale.

A principal component analysis of the chemical monitoring data explained 41% of the variance across the sampled species and sites (Fig. 6a). The analysis showed that *P. ulvae* and *H. diversicolor* were most impacted in terms of chemical body burden at Hythe with *G. pulex* clustering more closely with the site clusters for Wivenhoe and Alresford. The closer association of *G. pulex* with these two downstream sites is attributed to the lower chemical burden in biota that were sampled from them. Interestingly, the confidence ellipses are clustered within each other and indicate that variance in occurrence data is smaller the further samples are taken from the point source. Contamination was generally low with (semi)solid samples (i.e., sediment and biota) on the parts per billion scale and surface water samples in parts per trillion.

Comparing the average contamination for each compartment (sediment, water & biota) showed that sediment had a higher contaminant burden followed by biota and then surface water which is an order of magnitude lower (Fig. 6b). The mean chemical burden for sediment samples was 13 ± 24, 6 ± 10 and 5 ± 6 ng g^−1^. In comparison, the average chemical burden in biota samples was 6 ± 11, 3 ± 5 and 3 ± 4 ng g^−1^ and in surface water samples was 52 ± 73, 8 ± 12, 7 ± 12 ng L^−1^ for Hythe, Wivenhoe and Alresford, respectively. Reduction in the mean burden from the first site (Hythe) to second site (Wivenhoe) for sediment was 2.2-fold, 1.6-fold in biota and 6.2-fold in surface water. The greater reduction in surface water is likely to arise from multiple processes occurring including dilution (rainfall/tide), degradation, transformation, sorption and accumulation. Interestingly, fewer compounds were determined in both sediment and biota samples when compared with surface water which has been seen in previous multi-compartmental studies (Inostroza et al., 2017; Wilkinson et al., 2018). Therefore, it is more useful to measure multiple compartments in an aquatic habitat to gain a holistic understanding of exposure and arguably biomonitoring should be of primary focus in terms of relating pollutants to their potential risk in the wider environment.

## 4 Conclusion

A total of 70 unique compounds were determined across surface water, sediment and biota samples collected from the three sites along the estuarine River Colne (Essex, UK). The most frequently detected chemicals belonged to pharmaceuticals and recreational drugs. Of these, psychoactive pharmaceuticals showed the highest concentrations across all compartments including multiple macroinvertebrate species that are unique and important resources for estuarine birds and fishes. The data suggest that sediment is an important exposure route with benthic-dwelling organisms typically showing higher contaminant concentrations. Amphipods showed lower contamination and may indicate that these are a more conservative indicator species for environmental exposure. The neonicotinoid, imidacloprid was determined at higher concentrations in sediment and biota samples despite the recent EU-wide ban, although the source of contamination was unclear. Additional pesticides that no longer have approval for use in the EU were also detected which included fenuron, atrazine, pymetrozine and simazine. The detection of these banned substances is a cause for concern and further investigations should look to understand the source of the input. The mixture of chemicals present in the different compartments may be associated with potential hazards for organisms exposed to them. With this in mind, it is important to increase our surveillance in the environment so that we can identify areas and chemicals that are of higher concern. As a final consideration whilst broad targeted analytical methods are useful to quantitatively determine chemical contaminants the bias of these lists is problematic for characterising the full extent of the exposome. Thus, future studies should consider non-target exposomics-type strategies where possible and across multiple compartments to give greater coverage of the contaminant space in the aquatic environment.

## CRediT statement

**Thomas H. Miller**: Conceptualization, Methodology, Investigation, Software, Formal analysis, Data Curation, Writing - Original & Draft, Writing - Reviewing & Editing, Visualisation, Supervision, Funding Acquisition, Resources, Project Administration. **Keng Tiong Ng**: Formal analysis, Data Curation, Investigation, Writing - Original & Draft, Resources. **Aaron Lamphiere**: Methodology, Investigation. **Tom C. Cameron**: Conceptualization, Writing - Original & Draft, Supervision. **Nicolas R. Bury**: Conceptualization, Writing - Original & Draft, Resources, Project Administration, Investigation, Supervision. **Leon P. Barron**: Writing - Original & Draft, Supervision, Funding Acquisition, Project Administration.

## Declaration of competing interest

The authors declare that they have no known competing financial interests or personal relationships that could have appeared to influence the work reported in this paper.
