# Supplementary material for: Multicompartment and cross-species monitoring of contaminants of emerging concern in an estuarine habitat
Source: Environ Pollut. 2021 Feb 1;270:116300. doi: 10.1016/j.envpol.2020.116300 (PMC7846722; doi:10.1016/j.envpol.2020.116300)
Supplement: Multimedia component 1 [file mmc1.docx]

*Supplementary Information*

**MULTICOMPARTMENT AND CROSS-SPECIES MONITORING OF CONTAMINANTS OF EMERGING CONCERN IN AN ESTUARINE HABITAT**

*Thomas H. Miller^ab^*, Keng Tiong Ng^bf^, Aaron Lamphiere^c^, Tom C. Cameron^c†^, Nicolas R. Bury^d^,^e†^, Leon P. Barron^bf†^*

*^a^Department of Life Sciences, College of Health and Life Sciences, Brunel University London, Kingston Lane, UB8 3PH, UK.*

*^b^Department of Analytical, Environmental & Forensic Sciences, School of Population Health & Environmental Sciences, Faculty of Life Sciences and Medicine, King’s College London, 150 Stamford Street, London, SE1 9NH, UK.*

*^c^School of Life Sciences, University of Essex, Wivenhoe Park, Colchester, Essex, CO43SQ*

*^d^School of Science, Technology and Engineering, University of Suffolk, James Hehir Building, University Avenue, Ipswich, Suffolk, IP3 0FS, UK.*

*^e^Suffolk Sustainability, University of Suffolk, Waterfront Building, Neptune Quay, Ipswich, IP4 1QJUK.*

*^f^Environmental Research Group, School of Public Health, Faculty of Medicine, Imperial College London, UK*

*†Principal Investigators*

**Corresponding author*

*E-mail: thomas.miller@brunel.ac.uk*

Table of Contents

**S1.0** Analytical Standards……………………………………………………………....……S3

**S2.0** Instrumental Conditions…………………………………………………………...……S4

**S3.0** Comparison of compartmental contamination……………….……………………..S15

*List of Tables*

**Table S1.** Overview of mass spectrometer conditions

**Table S2.** MRM transitions for target analytes and SIL-IS.

**Table S3.** Method repeatability for sediment extraction and analysis at two concentrations (n=5).

**Table S4.** SIL-IS correction applied for the specified analytes that were detected in the present study. SIL-IS were not applied to analytes that demonstrated acceptable linearity (R^2^>0.98)

*List of Figures*

**Figure S1.** Venn diagram showing a comparison of the number of chemicals detected across the three compartments analysed in the Colne estuary including sediment, surface water and biota. Biota includes all macroinvertebrate species sampled.

**S1. Analytical Standards**

Reference standards for metformin-HCl, amphetamine, salicylic acid, dimetridazole, methamphetamine, nicotine, methcathinone, fenuron, cyromazine, cotinine, mephedrone, memantine-HCl, 4-fluoromethcathinone, propamocarb, antipyrine, 4-methylethcathinone, methedrone, 3,4-Methylenedioxymethamphetamine (MDMA), cymoxanil, cycluron, tacrine, ronidazole, simazine, levamisole-HCl, ibuprofen, methylone, ethirimol, clofibric acid, atrazine, oxamyl, acetamiprid, prometon, ametryn, propazine, diuron, methylphenidate-HCl, lidocaine, carboxine, carbamazepine, ketamine, mefenamic acid, bupropion-HCl, salbutamol, gemfibrozil, clothiandin, sulfapyridine, sulfadiazine, carbamazepine-10,11-epoxide, thiacloprid, sulfamethoxazole, ketoprofen, sulfathiazole, imidacloprid, dimethametryn, diphenhydramine-HCl, propranolol, tramadol-HCl, nortriptyline, sulfamerazine, aclonifen, sulfisoxazole, oxycarboxine, metoprolol, orphenadrine, mephosfolan, rizatriptan, nitenpyram, nordiazepam, flutamide, clotrimazole, venlafaxine-HCl, amitriptyline, sulfamethazine, sulfamonomethoxine, diazepam, morphine, oxazepam, ethofumesate, benzoylecgonine (BZE), thiamethoxam, diclofenac, meclofenamic acid, hydrochlorothiazide, norfluoxetine, carazolol, norethisterone, temazepam, cocaine, sertraline, warfarin, betaxolol, benzotropine, alprazolam, nadolol, fluoxetine, ketotifen, sulfadimethoxine, levonorgestrel, oxycodone-HCl, timolol, cocaethylene, chloropromazine-HCl, norfloxacin, lorazepam, clopidogrel-HSO4^-^, pyripoxyfen, flutolanil, citalopram-HBr, bisoprolol, cycloxidim, clozapine, paroxetine-HCl, nifedipine, medroxyprogesterone, prodiamine, lomefloxacin-HCl, pirenzepine-diHCL, piperophos, indomethacin, bezafibrate, fenofibrate, ofloxacine, enrofloxacin, fenoxaprop-p-ethyl, picoxystrobin, isradipine, fleroxacin, famoxadone, enalapril, 6a-methylprednisolone, haloperidol, cyphenothrin, celecoxib, sarafloxacin-HCl, busipirone-HCl, pyraclostrobin, dimethomorph, meclizine-HCl, bensulide, thiazopyr, difloxacin-HCl, azoxystrobin, lincomycin-HCl, amlodipine, tamsulosin-HCl, risperidone, carfentrazone-ethyl, ziprasidone-HCl, miconazole, cilazapril, levocabastine-HCl, valsartan, verapamil-HCl, oxytetracycline-HCl, terfenadine, chlortetracycline, flufenoxuron, fluocinide, ketoconazole, atorvastatin, azelnidipine, amiodarone-HCl, spinosad A, spinosad D, clarithromycin, azithromycin, josamycin and spiramycin were all obtained from Sigma-Aldrich (Steinheim, Germany). Trimethoprim, caffeine, and naproxen were ordered from Fluka (Buchs, Switzerland). Stable isotope-labelled standards (SIL-IS) including amphetamine-d6, nicotine-d4, cotinine-d3, MDMA-d5, methylone-d3-HCl, ketamine-d4-HCl, notriptyline-d3-HCl, tramadol-^13^C1,d3-HCl, nordiazepam-d5, amitriptyline-d3-HCl, venlafaxine-d6-HCl, diazepam-d6, oxazepam-d5, BZE-d3, norfluoxetine-d6, temazepam-d5, cocaine-d3, sertraline-d3-HCl, fluoxetine-d6, lorazepam-d4, haloperidol-d4, risperidone-d4, clothiandin-d3 and thiamethoxam-d3 were ordered from Sigma-Aldrich. Celecoxib-d7, clarithromycin-d3, betaxolol-d7-HCl, cetirizine-d4, gemfibrozil-d6, metoprolol-d7-HCl, lidocaine-d10-HCl, nifedipine-d4, sulfamethazine-d4, verapamil-d3-HCl and trimethoprim-d3 were sourced from QMX (Essex, UK).

**S2. Instrumental Conditions**

**Table S1:** Overview of mass spectrometer conditions.

| ESI interface conditions | |
| --- | --- |
| Nebulising gas flow, L/min | 3 |
| Heating gas flow, L/min | 10 |
| Interface temperature, °C | 300 |
| DL temperature, °C | 250 |
| Heat block temperature, °C | 400 |
| Drying gas flow, L/min | 10 |

**Table S2:** MRM transitions for target analytes and SIL-IS.

| **Compounds** | **Precursor**  **(m/z)** | **Transition (m/z)** | | **Polarity** | | **Pause Time (ms)** | **Dwell Time (ms)** | **Q1 Pre Bias**  **(V)** | **Collision Energy (V)** | | **Q3 Pre Bias**  **(V)** |
| --- | --- | --- | --- | --- | --- | --- | --- | --- | --- | --- | --- |
| 2-(Thiocyanomethylthio)benzothiazole | 238.7 | | 180.1 | | + | 1 | 4 | -27 | | -14 | -19 |
|  |  | | 136.1 | | + | 1 | 4 | -29 | | -26 | -14 |
| 4-Fluoromethcathinone | 182.1 | | 164.2 | | + | 1 | 5 | -30 | | -15 | -17 |
|  |  | | 149.1 | | + | 1 | 5 | -13 | | -22 | -27 |
| 4-Methylethcathinone | 192.1 | | 174.2 | | + | 1 | 5 | -23 | | -16 | -18 |
|  |  | | 144.1 | | + | 1 | 5 | -10 | | -29 | -25 |
| Acetamiprid | 233.0 | | 126.1 | | + | 1 | 12 | -16 | | -21 | -13 |
| Alprazolam | 309.1 | | 281.1 | | + | 1 | 4 | -16 | | -26 | -22 |
|  |  | | 205.1 | | + | 1 | 4 | -16 | | -40 | -23 |
| Ametryn | 228.1 | | 186.1 | | + | 1 | 4 | -11 | | -20 | -20 |
|  |  | | 96.1 | | + | 1 | 4 | -23 | | -28 | -19 |
| Amiodarone | 645.8 | | 58.2 | | + | 1 | 71 | -32 | | -49 | -27 |
|  |  | | 100.2 | | + | 1 | 71 | -32 | | -30 | -10 |
| Amitriptyline | 278.2 | | 91.1 | | + | 1 | 5 | -13 | | -27 | -19 |
|  |  | | 105.1 | | + | 1 | 5 | -13 | | -25 | -21 |
| Amitriptyline-d3 | 281.1 | | 233.2 | | + | 1 | 5 | -19 | | -17 | -25 |
|  |  | | 105.2 | | + | 1 | 5 | -10 | | -23 | -19 |
| Amlodipine | 409.1 | | 238.2 | | + | 1 | 10 | -15 | | -12 | -24 |
| Amphetamine | 136.1 | | 91.1 | | + | 1 | 5 | -14 | | -17 | -19 |
|  |  | | 119.1 | | + | 1 | 5 | -14 | | -15 | -25 |
| Amphetamine-d6 | 142.2 | | 93.2 | | + | 1 | 5 | -17 | | -19 | -17 |
|  |  | | 125.2 | | + | 1 | 5 | -16 | | -14 | -21 |
| Antipyrine | 188.9 | | 77.2 | | + | 1 | 5 | -13 | | -40 | -30 |
|  |  | | 56.2 | | + | 1 | 5 | -13 | | -32 | -23 |
| Atorvastatin | 559.1 | | 440.3 | | + | 1 | 6 | -20 | | -23 | -16 |
|  |  | | 250.2 | | + | 1 | 6 | -20 | | -43 | -27 |
| Atrazine | 216.1 | | 174.1 | | + | 1 | 14 | -11 | | -18 | -18 |
| Azelnidipine | 583.2 | | 167.2 | | + | 1 | 4 | -40 | | -27 | -30 |
|  |  | | 165.2 | | + | 1 | 4 | -40 | | -55 | -16 |
| Azithromycin | 749.6 | | 591.4 | | + | 1 | 4 | -28 | | -30 | -22 |
|  |  | | 158.3 | | + | 1 | 4 | -28 | | -40 | -16 |
| Azoxystrobin | 404.2 | | 372.1 | | + | 1 | 4 | -21 | | -16 | -27 |
|  |  | | 344.1 | | + | 1 | 4 | -21 | | -26 | -24 |
| Benoxacor | 260.0 | | 149.2 | | + | 1 | 4 | -30 | | -18 | -27 |
|  |  | | 134.1 | | + | 1 | 4 | -30 | | -29 | -25 |
| Bensulide | 398.1 | | 158.1 | | + | 1 | 4 | -20 | | -24 | -16 |
|  |  | | 217.9 | | + | 1 | 4 | -20 | | -17 | -23 |
| Benzatropine | 308.1 | | 167.2 | | + | 1 | 6 | -11 | | -30 | -17 |
|  |  | | 165.2 | | + | 1 | 6 | -21 | | -52 | -17 |
| Benzoylecgonine, BZE | 290.1 | | 168.2 | | + | 1 | 12 | -20 | | -19 | -18 |
| Benzoylecgonine, BZE-d3 | 293.1 | | 171.2 | | + | 1 | 12 | -30 | | -20 | -18 |
| Betaxolol | 308.3 | | 116.2 | | + | 1 | 4 | -15 | | -21 | -21 |
|  |  | | 72.2 | | + | 1 | 4 | -21 | | -24 | -27 |
| Betaxolol-d7 | 315.2 | | 123.3 | | + | 1 | 4 | -23 | | -22 | -22 |
|  |  | | 105.3 | | + | 1 | 4 | -23 | | -24 | -11 |
| Bezafibrate | 360.2 | | 274.0 | | - | 1 | 5 | 18 | | 17 | 12 |
|  |  | | 154.1 | | - | 1 | 5 | 20 | | 29 | 15 |
| Bisoprolol | 326.2 | | 116.2 | | + | 1 | 6 | -23 | | -19 | -21 |
|  |  | | 74.2 | | + | 1 | 6 | -12 | | -26 | -29 |
| Bupropion | 240.1 | | 184.2 | | + | 1 | 4 | -29 | | -13 | -12 |
|  |  | | 131.2 | | + | 1 | 4 | -27 | | -26 | -13 |
| Buspirone | 386.1 | | 122.2 | | + | 1 | 4 | -26 | | -30 | -23 |
|  |  | | 109.2 | | + | 1 | 4 | -25 | | -46 | -11 |
| Carazolol | 299.1 | | 116.2 | | + | 1 | 4 | -21 | | -21 | -20 |
|  |  | | 222.2 | | + | 1 | 4 | -21 | | -21 | -24 |
| Carbamazepine | 237.1 | | 194.0 | | + | 1 | 6 | -12 | | -20 | -23 |
|  |  | | 192.1 | | + | 1 | 6 | -12 | | -25 | -22 |
| Carboxine | 236.0 | | 143.1 | | + | 1 | 12 | -26 | | -15 | -14 |
| Carfentrazone-ethyl | 412.1 | | 346.0 | | + | 1 | 4 | -19 | | -17 | -24 |
|  |  | | 366.0 | | + | 1 | 4 | -19 | | -19 | -21 |
| Carbamazepine-10,11-epoxide | 252.9 | | 180.2 | | + | 1 | 5 | -17 | | -29 | -18 |
|  |  | | 236.2 | | + | 1 | 5 | -17 | | -12 | -16 |
| Celecoxib | 382.1 | | 362.1 | | + | 1 | 4 | -11 | | -28 | -25 |
|  |  | | 300.2 | | + | 1 | 4 | -18 | | -28 | -21 |
| Celecoxib-d7 | 389.0 | | 369.1 | | + | 1 | 4 | -19 | | -29 | -25 |
|  |  | | 289.2 | | + | 1 | 4 | -26 | | -37 | -30 |
| Cetrizine-d4 | 393.1 | | 201.1 | | + | 1 | 6 | -20 | | -25 | -21 |
|  |  | | 165.2 | | + | 1 | 6 | -20 | | -53 | -26 |
| Chloramphenicol | 322.7 | | 152.2 | | - | 1 | 5 | 16 | | 17 | 15 |
|  |  | | 257.0 | | - | 1 | 5 | 17 | | 11 | 25 |
| Cilazapril | 418.0 | | 211.2 | | + | 1 | 5 | -20 | | -20 | -20 |
|  |  | | 70.2 | | + | 1 | 5 | -15 | | -46 | -12 |
| Citalopram | 325.1 | | 109.2 | | + | 1 | 4 | -22 | | -26 | -20 |
|  |  | | 262.2 | | + | 1 | 4 | -24 | | -20 | -30 |
| Clarithromycin | 748.2 | | 158.2 | | + | 1 | 4 | -36 | | -31 | -10 |
|  |  | | 590.4 | | + | 1 | 4 | -36 | | -20 | -22 |
| Clarithromycin-d3 | 751.2 | | 161.2 | | + | 1 | 4 | -38 | | -28 | -16 |
|  |  | | 593.4 | | + | 1 | 4 | -38 | | -22 | -22 |
| Clodinafop-propargyl | 350.1 | | 266.1 | | + | 1 | 4 | -18 | | -17 | -19 |
|  |  | | 238.2 | | + | 1 | 4 | -18 | | -27 | -24 |
| Clofibric acid | 212.5 | | 126.9 | | - | 1 | 12 | 11 | | 15 | 24 |
| Clothianidin | 250.1 | | 132.0 | | + | 1 | 5 | -12 | | -15 | -13 |
|  |  | | 169.1 | | + | 1 | 5 | -12 | | -13 | -18 |
| Clothianidin-d3 | 254.6 | | 172.2 | | + | 1 | 5 | -27 | | -13 | -17 |
|  |  | | 134.1 | | + | 1 | 5 | -12 | | -17 | -24 |
| Clozapine | 327.2 | | 270.2 | | + | 1 | 4 | -16 | | -23 | -20 |
|  |  | | 192.1 | | + | 1 | 4 | -16 | | -45 | -14 |
| Cocaine | 304.2 | | 182.3 | | + | 1 | 6 | -11 | | -21 | -14 |
|  |  | | 82.1 | | + | 1 | 6 | -11 | | -35 | -17 |
| Cocaine-d3 | 307.1 | | 185.2 | | + | 1 | 6 | -22 | | -21 | -19 |
|  |  | | 77.2 | | + | 1 | 6 | -15 | | -54 | -14 |
| Cotinine-d3 | 179.9 | | 80.2 | | + | 1 | 5 | -30 | | -26 | -15 |
|  |  | | 101.2 | | + | 1 | 5 | -30 | | -22 | -18 |
| Cyclouron | 199.1 | | 72.2 | | + | 1 | 6 | -14 | | -25 | -13 |
|  |  | | 89.2 | | + | 1 | 6 | -22 | | -14 | -16 |
| Cycloxyidim | 326.1 | | 280.2 | | + | 1 | 6 | -16 | | -14 | -19 |
|  |  | | 180.2 | | + | 1 | 6 | -12 | | -22 | -19 |
| Cymoxanil | 199.2 | | 111.2 | | + | 1 | 6 | -23 | | -19 | -18 |
|  |  | | 83.0 | | + | 1 | 6 | -23 | | -27 | -17 |
| Cyphenothrin | 376.2 | | 181.0 | | + | 1 | 4 | -11 | | -28 | -20 |
|  |  | | 151.1 | | + | 1 | 4 | -11 | | -11 | -25 |
| Cyromazine | 167.2 | | 68.0 | | + | 1 | 9 | -19 | | -32 | -26 |
|  |  | | 59.9 | | + | 1 | 9 | -19 | | -22 | -24 |
| Diazepam | 285.1 | | 154.1 | | + | 1 | 4 | -11 | | -28 | -18 |
|  |  | | 193.1 | | + | 1 | 4 | -11 | | -29 | -23 |
| Diazepam-d6 | 289.9 | | 198.2 | | + | 1 | 4 | -19 | | -33 | -21 |
|  |  | | 154.2 | | + | 1 | 4 | -19 | | -27 | -15 |
| Diclofenac | 296.0 | | 215.1 | | + | 1 | 6 | -15 | | -20 | -16 |
|  |  | | 214.0 | | + | 1 | 6 | -15 | | -40 | -24 |
| Diflubenzuron | 311.0 | | 158.1 | | + | 1 | 4 | -16 | | -16 | -29 |
|  |  | | 141.1 | | + | 1 | 4 | -16 | | -32 | -27 |
| Dimethametryn | 256.1 | | 186.2 | | + | 1 | 4 | -28 | | -22 | -30 |
|  |  | | 68.1 | | + | 1 | 4 | -13 | | -44 | -11 |
| Dimethomorph | 388.2 | | 301.1 | | + | 1 | 4 | -20 | | -21 | -21 |
|  |  | | 165.1 | | + | 1 | 4 | -20 | | -35 | -17 |
| Diphenhydramine | 256.0 | | 167.1 | | + | 1 | 4 | -30 | | -11 | -12 |
|  |  | | 152.0 | | + | 1 | 4 | -30 | | -40 | -17 |
| Disulfoton sulfone | 307.0 | | 97.0 | | + | 1 | 6 | -16 | | -30 | -17 |
|  |  | | 125.1 | | + | 1 | 6 | -16 | | -18 | -23 |
| Diuron (DCMU) | 231.0 | | 186.0 | | - | 1 | 6 | 16 | | 19 | 20 |
|  |  | | 150.0 | | - | 1 | 6 | 16 | | 25 | 30 |
| Enalapril | 375.1 | | 114.0 | | - | 1 | 12 | 27 | | 24 | 21 |
| Ethirimol | 210.2 | | 98.1 | | + | 1 | 5 | -25 | | -22 | -10 |
|  |  | | 128.2 | | + | 1 | 5 | -27 | | -11 | -28 |
| Ethofumesate | 287.5 | | 247.1 | | + | 1 | 12 | -13 | | -16 | -12 |
| Famoxadone | 375.8 | | 196.1 | | + | 1 | 10 | -25 | | -21 | -20 |
| Fenoxaprop-ethyl | 362.1 | | 288.1 | | + | 1 | 4 | -18 | | -18 | -21 |
|  |  | | 121.1 | | + | 1 | 4 | -19 | | -33 | -13 |
| Fenuron | 165.0 | | 72.2 | | + | 1 | 5 | -18 | | -22 | -28 |
|  |  | | 46.1 | | + | 1 | 5 | -18 | | -14 | -18 |
| Flufenoxuron | 489.1 | | 158.1 | | + | 1 | 4 | -15 | | -21 | -17 |
|  |  | | 141.0 | | + | 1 | 4 | -24 | | -39 | -15 |
| Fluocinonide | 494.9 | | 337.2 | | + | 1 | 10 | -23 | | -19 | -25 |
| Fluoxetine | 310.2 | | 44.2 | | + | 1 | 4 | -15 | | -16 | -17 |
|  |  | | 148.2 | | + | 1 | 4 | -15 | | -10 | -11 |
| Fluoxetine-d6 | 316.1 | | 44.2 | | + | 1 | 4 | -20 | | -15 | -20 |
|  |  | | 154.3 | | + | 1 | 4 | -15 | | -9 | -16 |
| Flurbiprofen | 245.2 | | 188.7 | | + | 1 | 32 | -24 | | -15 | -29 |
| Flurochloridone | 312.0 | | 291.9 | | + | 1 | 4 | -16 | | -21 | -20 |
|  |  | | 144.9 | | + | 1 | 4 | -16 | | -46 | -29 |
| Flutamide | 275.0 | | 202.0 | | - | 1 | 4 | 14 | | 23 | 20 |
|  |  | | 205.0 | | - | 1 | 4 | 13 | | 21 | 23 |
| Flutolanil | 324.3 | | 242.2 | | + | 1 | 4 | -15 | | -26 | -16 |
|  |  | | 262.2 | | + | 1 | 4 | -15 | | -19 | -18 |
| Fuberidazole | 184.9 | | 157.2 | | + | 1 | 5 | -23 | | -21 | -16 |
|  |  | | 156.2 | | + | 1 | 5 | -23 | | -27 | -16 |
| Gemfibrozil | 249.2 | | 121.2 | | - | 1 | 10 | 28 | | 25 | 11 |
| Gemfibrozil-d6 | 255.2 | | 121.1 | | - | 1 | 10 | 29 | | 22 | 20 |
| Haloperidol | 376.2 | | 123.1 | | + | 1 | 4 | -18 | | -42 | -26 |
|  |  | |  | | + | 1 | 4 | -18 | | -25 | -12 |
| Haloperidol-d4 | 380.2 | | 169.2 | | + | 1 | 4 | -25 | | -26 | -17 |
|  |  | |  | | + | 1 | 4 | -25 | | -40 | -21 |
| Hydrochlorothiazide | 296.0 | | 269.0 | | - | 1 | 12 | 11 | | 19 | 11 |
| Ibuprofen | 205.2 | | 161.2 | | - | 1 | 12 | 22 | | 9 | 17 |
| Imidacloprid | 256.1 | | 175.1 | | + | 1 | 5 | -13 | | -19 | -20 |
|  |  | | 209.1 | | + | 1 | 5 | -13 | | -18 | -10 |
| Indomethacin | 356.5 | | 312.3 | | - | 1 | 14 | 18 | | 11 | 10 |
| Isocarbamid | 186.1 | | 87.0 | | + | 1 | 5 | -20 | | -16 | -15 |
|  |  | | 44.2 | | + | 1 | 5 | -20 | | -32 | -17 |
| Isradipine | 370.2 | | 119.0 | | - | 1 | 4 | 14 | | 16 | 11 |
|  |  | | 250.0 | | - | 1 | 4 | 11 | | 16 | 17 |
| Josamycin | 828.5 | | 109.3 | | + | 1 | 10 | -30 | | -47 | -27 |
| Ketamine | 238.1 | | 125.0 | | + | 1 | 6 | -12 | | -26 | -26 |
|  |  | | 207.0 | | + | 1 | 6 | -12 | | -15 | -15 |
| Ketamine-d4 | 242.1 | | 129.2 | | + | 1 | 6 | -25 | | -28 | -26 |
|  |  | | 211.3 | | + | 1 | 6 | -28 | | -15 | -10 |
| Ketoconazole | 533.0 | | 491.1 | | + | 1 | 4 | -36 | | -31 | -24 |
|  |  | | 82.2 | | + | 1 | 4 | -36 | | -47 | -14 |
| Ketoprofen | 253.1 | | 209.1 | | - | 1 | 5 | 23 | | 8 | 20 |
|  |  | | 221.1 | | - | 1 | 5 | 13 | | 17 | 14 |
| Ketotifen | 310.0 | | 96.2 | | + | 1 | 10 | -22 | | -24 | -18 |
| Levamisole | 207.1 | | 180.0 | | + | 1 | 5 | -10 | | -24 | -20 |
|  |  | | 91.1 | | + | 1 | 5 | -10 | | -41 | -17 |
| Levocabastine | 421.4 | | 174.2 | | + | 1 | 6 | -20 | | -32 | -18 |
|  |  | | 70.2 | | + | 1 | 6 | -15 | | -37 | -12 |
| Levonorgestrel | 313.1 | | 245.3 | | + | 1 | 4 | -22 | | -18 | -27 |
|  |  | | 109.2 | | + | 1 | 4 | -21 | | -27 | -20 |
| Lidocaine | 235.0 | | 86.1 | | + | 1 | 6 | -26 | | -19 | -18 |
|  |  | | 58.1 | | + | 1 | 6 | -26 | | -45 | -12 |
| Lidocaine-d10 | 245.2 | | 96.3 | | + | 1 | 14 | -29 | | -22 | -17 |
| Lincomycin | 407.2 | | 126.2 | | + | 1 | 5 | -22 | | -31 | -13 |
|  |  | | 359.2 | | + | 1 | 5 | -23 | | -19 | -27 |
| Lorazepam | 321.1 | | 303.0 | | + | 1 | 6 | -12 | | -10 | -24 |
|  |  | | 274.9 | | + | 1 | 6 | -12 | | -22 | -22 |
|  |  | |  | |  |  |  |  | |  |  |
| Lorazepam-d4 | 325.1 | | 279.1 | | + | 1 | 6 | -22 | | -23 | -19 |
|  |  | | 307.1 | | + | 1 | 6 | -23 | | -17 | -21 |
| MDMA | 194.1 | | 163.1 | | + | 1 | 5 | -10 | | -13 | -12 |
|  |  | | 105.1 | | + | 1 | 5 | -10 | | -25 | -22 |
| MDMA-d5 | 199.1 | | 165.3 | | + | 1 | 5 | -22 | | -14 | -29 |
|  |  | | 107.2 | | + | 1 | 5 | -10 | | -25 | -11 |
| Meclizine | 391.1 | | 201.1 | | + | 1 | 4 | -28 | | -20 | -22 |
|  |  | | 165.1 | | + | 1 | 4 | -19 | | -55 | -17 |
| Medroxyprogesterone | 345.1 | | 123.2 | | + | 1 | 4 | -23 | | -26 | -21 |
|  |  | | 97.2 | | + | 1 | 4 | -23 | | -27 | -10 |
| Mefenamic acid | 240.0 | | 196.2 | | - | 1 | 14 | 17 | | 18 | 12 |
| Memantine | 180.3 | | 163.3 | | + | 1 | 6 | -19 | | -18 | -17 |
|  |  | | 107.3 | | + | 1 | 6 | -20 | | -26 | -20 |
| Mephedrone | 178.3 | | 145.1 | | + | 1 | 5 | -20 | | -21 | -29 |
|  |  | | 160.3 | | + | 1 | 5 | -20 | | -16 | -11 |
| Mephosfolan | 270.1 | | 140.0 | | + | 1 | 14 | -13 | | -25 | -14 |
| Metformin | 130.3 | | 71.2 | | + | 1 | 11 | -23 | | -23 | -12 |
|  |  | | 60.2 | | + | 1 | 11 | -14 | | -15 | -26 |
| Methamphetamine | 150.1 | | 91.1 | | + | 1 | 5 | -10 | | -19 | -18 |
|  |  | | 119.1 | | + | 1 | 5 | -10 | | -10 | -24 |
| Methcathinone | 164.0 | | 131.2 | | + | 1 | 5 | -29 | | -21 | -25 |
|  |  | | 146.2 | | + | 1 | 5 | -11 | | -16 | -27 |
| Methedrone | 194.3 | | 176.2 | | + | 1 | 5 | -20 | | -15 | -29 |
|  |  | | 161.1 | | + | 1 | 5 | -21 | | -21 | -16 |
| Methylone-d3 | 211.1 | | 163.2 | | + | 1 | 5 | -24 | | -17 | -28 |
|  |  | | 135.2 | | + | 1 | 5 | -24 | | -27 | -13 |
| Methylphenidate | 234.2 | | 84.1 | | + | 1 | 6 | -11 | | -20 | -17 |
|  |  | | 56.1 | | + | 1 | 6 | -11 | | -45 | -22 |
| Methylphenidate-d9 | 243.3 | | 93.3 | | + | 1 | 6 | -28 | | -24 | -17 |
|  |  | | 61.2 | | + | 1 | 6 | -27 | | -50 | -23 |
| Metoprolol | 268.2 | | 116.2 | | + | 1 | 5 | -30 | | -21 | -21 |
|  |  | | 159.1 | | + | 1 | 5 | -10 | | -22 | -16 |
| Metoprolol-d7 | 275.2 | | 123.2 | | + | 1 | 5 | -22 | | -21 | -23 |
|  |  | | 105.2 | | + | 1 | 5 | -29 | | -22 | -19 |
| Morphine | 286.2 | | 165.1 | | + | 1 | 4 | -14 | | -44 | -12 |
|  |  | | 153.1 | | + | 1 | 4 | -14 | | -45 | -29 |
| Morphine-d3 | 289.0 | | 152.2 | | + | 1 | 4 | -14 | | -55 | -30 |
|  |  | | 201.2 | | + | 1 | 4 | -14 | | -27 | -21 |
| Nadolol | 310.3 | | 254.2 | | + | 1 | 5 | -21 | | -17 | -17 |
|  |  | | 236.2 | | + | 1 | 5 | -21 | | -21 | -25 |
| Naproxen | 229.1 | | 169.3 | | - | 1 | 12 | 18 | | 29 | 19 |
| Nicotine | 163.0 | | 117.2 | | + | 1 | 5 | -20 | | -30 | -20 |
|  |  | | 130.2 | | + | 1 | 5 | -20 | | -25 | -20 |
| Nicotine-d4 | 167.0 | | 134.2 | | + | 1 | 5 | -12 | | -22 | -25 |
|  |  | | 121.2 | | + | 1 | 5 | -18 | | -28 | -21 |
| Nifedipine | 345.2 | | 222.1 | | - | 1 | 4 | 10 | | 10 | 15 |
|  |  | | 122.0 | | - | 1 | 4 | 13 | | 12 | 27 |
| Nifedipine-d4 | 349.0 | | 222.1 | | - | 1 | 4 | 10 | | 10 | 24 |
|  |  | | 126.1 | | - | 1 | 4 | 13 | | 12 | 28 |
| Nitenpyram | 271.0 | | 225.2 | | + | 1 | 5 | -19 | | -12 | -15 |
|  |  | | 126.2 | | + | 1 | 5 | -19 | | -28 | -13 |
| Nordiazepam | 270.8 | | 140.2 | | + | 1 | 4 | -18 | | -28 | -14 |
|  |  | | 208.1 | | + | 1 | 4 | -10 | | -28 | -14 |
| Nordiazepam-d5 | 275.9 | | 213.3 | | + | 1 | 4 | -10 | | -28 | -22 |
|  |  | | 140.2 | | + | 1 | 4 | -10 | | -31 | -14 |
| Norethisterone | 299.1 | | 109.2 | | + | 1 | 4 | -21 | | -26 | -19 |
|  |  | | 231.2 | | + | 1 | 4 | -11 | | -20 | -11 |
| Nortriptyline | 264.2 | | 233.1 | | + | 1 | 4 | -10 | | -15 | -17 |
|  |  | | 91.1 | | + | 1 | 4 | -10 | | -25 | -18 |
| Nortriptyline-d3 | 267.0 | | 233.2 | | + | 1 | 4 | -18 | | -15 | -16 |
|  |  | | 105.2 | | + | 1 | 4 | -18 | | -22 | -19 |
| Orphenadrine | 270.1 | | 181.2 | | + | 1 | 4 | -29 | | -13 | -29 |
|  |  | | 166.1 | | + | 1 | 4 | -10 | | -28 | -30 |
| Oxamyl | 237.1 | | 72.1 | | + | 1 | 5 | -12 | | -12 | -13 |
|  |  | | 90.2 | | + | 1 | 5 | -12 | | -10 | -17 |
| Oxazepam | 287.2 | | 241.2 | | + | 1 | 6 | -11 | | -24 | -19 |
|  |  | | 269.1 | | + | 1 | 6 | -11 | | -15 | -22 |
| Oxazepam-d5 | 292.0 | | 246.2 | | + | 1 | 6 | -22 | | -24 | -26 |
|  |  | | 274.1 | | + | 1 | 6 | -11 | | -17 | -13 |
| Oxycarboxin | 268.1 | | 175.0 | | + | 1 | 5 | -13 | | -14 | -18 |
|  |  | | 147.0 | | + | 1 | 5 | -13 | | -27 | -30 |
| Oxycodone | 316.2 | | 298.2 | | + | 1 | 11 | -16 | | -19 | -22 |
|  |  | | 241.1 | | + | 1 | 11 | -16 | | -30 | -18 |
| Picoxystrobin | 368.0 | | 145.2 | | + | 1 | 4 | -25 | | -22 | -15 |
|  |  | | 205.2 | | + | 1 | 4 | -25 | | -10 | -14 |
| Piperophos | 353.9 | | 171.1 | | + | 1 | 4 | -23 | | -22 | -18 |
|  |  | | 255.1 | | + | 1 | 4 | -22 | | -14 | -12 |
| Pirenzipine | 352.1 | | 113.3 | | + | 1 | 5 | -26 | | -22 | -22 |
|  |  | | 70.2 | | + | 1 | 5 | -10 | | -46 | -12 |
| Pretilachlor | 312.2 | | 252.1 | | + | 1 | 4 | -16 | | -17 | -17 |
|  |  | | 176.1 | | + | 1 | 4 | -16 | | -29 | -18 |
| Prodiamine | 349.2 | | 232.0 | | - | 1 | 4 | 10 | | 24 | 10 |
|  |  | | 216.0 | | - | 1 | 4 | 13 | | 29 | 14 |
| Prometon | 226.1 | | 184.2 | | + | 1 | 6 | -11 | | -20 | -12 |
|  |  | | 142.2 | | + | 1 | 6 | -11 | | -23 | -14 |
| Prometryn | 242.2 | | 158.1 | | + | 1 | 4 | -12 | | -25 | -16 |
|  |  | | 200.0 | | + | 1 | 4 | -12 | | -20 | -22 |
| Propamocarb | 189.2 | | 102.1 | | + | 1 | 5 | -27 | | -17 | -20 |
|  |  | | 74.2 | | + | 1 | 5 | -10 | | -26 | -30 |
| Propanolol | 260.1 | | 116.2 | | + | 1 | 4 | -30 | | -19 | -21 |
|  |  | | 183.2 | | + | 1 | 4 | -10 | | -20 | -18 |
| Propazine | 230.1 | | 188.2 | | + | 1 | 4 | -26 | | -19 | -20 |
|  |  | | 146.1 | | + | 1 | 4 | -25 | | -24 | -25 |
| Pymetrozine | 218.1 | | 105.1 | | + | 1 | 5 | -11 | | -21 | -21 |
|  |  | | 79.0 | | + | 1 | 5 | -11 | | -45 | -15 |
| Pyracarbolid | 218.1 | | 125.1 | | + | 1 | 6 | -24 | | -18 | -24 |
|  |  | | 97.1 | | + | 1 | 6 | -24 | | -28 | -17 |
| Pyraclostrobin | 390.1 | | 194.1 | | + | 1 | 4 | -19 | | -15 | -21 |
|  |  | | 163.1 | | + | 1 | 4 | -19 | | -27 | -17 |
| Pyraflufen-ethyl | 413.0 | | 339.0 | | + | 1 | 4 | -21 | | -20 | -23 |
|  |  | | 253.1 | | + | 1 | 4 | -21 | | -35 | -26 |
| Pyridaben | 364.8 | | 147.2 | | + | 1 | 4 | -17 | | -25 | -15 |
|  |  | | 309.1 | | + | 1 | 4 | -18 | | -14 | -15 |
| Pyriproxyfen | 322.1 | | 96.1 | | + | 1 | 4 | -16 | | -16 | -18 |
|  |  | | 78.1 | | + | 1 | 4 | -16 | | -54 | -14 |
| Risperidone | 411.2 | | 191.1 | | + | 1 | 4 | -12 | | -31 | -23 |
|  |  | | 69.1 | | + | 1 | 4 | -12 | | -50 | -27 |
| Risperidone-d4 | 415.2 | | 195.2 | | + | 1 | 10 | -29 | | -30 | -21 |
| Rizatriptan | 270.1 | | 201.2 | | + | 1 | 5 | -10 | | -14 | -21 |
|  |  | | 158.2 | | + | 1 | 5 | -20 | | -21 | -16 |
| Ronidazole | 201.0 | | 140.2 | | + | 1 | 13 | -14 | | -13 | -14 |
| Roxithromycin | 837.3 | | 679.4 | | + | 1 | 4 | -24 | | -22 | -24 |
|  |  | | 158.1 | | + | 1 | 4 | -24 | | -34 | -16 |
| Salbutamol | 240.1 | | 148.2 | | + | 1 | 7 | -29 | | -20 | -28 |
|  |  | | 222.3 | | + | 1 | 7 | -12 | | -12 | -23 |
| Salicylic acid | 137.2 | | 93.1 | | - | 1 | 10 | 20 | | 20 | 16 |
|  |  | | 65.1 | | - | 1 | 10 | 20 | | 30 | 24 |
| Sertraline | 306.1 | | 159.0 | | + | 1 | 5 | -15 | | -26 | -18 |
|  |  | | 275.1 | | + | 1 | 5 | -15 | | -15 | -20 |
| Sertraline-d3 | 309.0 | | 159.1 | | + | 1 | 5 | -30 | | -28 | -30 |
|  |  | | 275.1 | | + | 1 | 5 | -11 | | -14 | -29 |
| Simazine | 202.1 | | 104.0 | | + | 1 | 5 | -23 | | -25 | -19 |
|  |  | | 68.1 | | + | 1 | 5 | -23 | | -32 | -27 |
| Spinosyn A | 732.6 | | 142.1 | | + | 1 | 6 | -22 | | -34 | -29 |
|  |  | | 98.0 | | + | 1 | 6 | -22 | | -40 | -19 |
| Spinosyn D | 746.6 | | 142.1 | | + | 1 | 6 | -22 | | -36 | -15 |
|  |  | | 98.0 | | + | 1 | 6 | -22 | | -40 | -19 |
| Spiramycin | 843.5 | | 174.2 | | + | 1 | 10 | -24 | | -36 | -11 |
| Sulfadimethoxine | 311.1 | | 156.0 | | + | 1 | 5 | -16 | | -20 | -17 |
|  |  | | 92.1 | | + | 1 | 5 | -16 | | -32 | -17 |
| Sulfamerazine | 265.1 | | 92.1 | | + | 1 | 5 | -13 | | -34 | -17 |
|  |  | | 156.0 | | + | 1 | 5 | -13 | | -17 | -16 |
| Sulfamethazine | 278.9 | | 186.1 | | + | 1 | 5 | -18 | | -17 | -19 |
|  |  | | 124.2 | | + | 1 | 5 | -10 | | -24 | -12 |
| Sulfamethazine-d4 | 282.8 | | 186.2 | | + | 1 | 5 | -13 | | -20 | -19 |
|  |  | | 124.2 | | + | 1 | 5 | -17 | | -25 | -27 |
| Sulfamethoxazole | 254.1 | | 156.0 | | + | 1 | 5 | -12 | | -18 | -16 |
|  |  | | 92.2 | | + | 1 | 5 | -12 | | -31 | -16 |
| Sulfamonomethoxine | 281.1 | | 156.1 | | + | 1 | 5 | -14 | | -18 | -17 |
|  |  | | 92.2 | | + | 1 | 5 | -14 | | -33 | -18 |
| Sulfapyridine | 250.0 | | 156.0 | | + | 1 | 5 | -23 | | -17 | -16 |
|  |  | | 92.1 | | + | 1 | 5 | -22 | | -32 | -18 |
| Sulfathiazole | 256.0 | | 156.0 | | + | 1 | 5 | -24 | | -16 | -16 |
|  |  | | 92.2 | | + | 1 | 5 | -24 | | -27 | -17 |
| Sulfisoxazole | 268.0 | | 156.1 | | + | 1 | 7 | -30 | | -15 | -16 |
|  |  | | 113.2 | | + | 1 | 7 | -10 | | -16 | -11 |
| Tacrine | 199.0 | | 171.2 | | + | 1 | 6 | -23 | | -30 | -17 |
|  |  | | 144.1 | | + | 1 | 6 | -14 | | -36 | -28 |
| Tamsulosin | 409.1 | | 228.1 | | + | 1 | 4 | -28 | | -24 | -24 |
|  |  | | 271.2 | | + | 1 | 4 | -28 | | -20 | -13 |
| Temazepam | 301.1 | | 255.1 | | + | 1 | 4 | -11 | | -25 | -30 |
|  |  | | 283.2 | | + | 1 | 4 | -11 | | -13 | -23 |
| Temazepam-d5 | 306.0 | | 260.1 | | + | 1 | 10 | -11 | | -24 | -17 |
| Terbutryn | 242.1 | | 186.1 | | + | 1 | 4 | -28 | | -20 | -20 |
|  |  | | 158.2 | | + | 1 | 4 | -29 | | -24 | -27 |
| Terfenadine | 472.4 | | 436.3 | | + | 1 | 10 | -24 | | -28 | -22 |
|  |  | | 454.3 | | + | 1 | 10 | -13 | | -22 | -22 |
| Thiacloprid | 253.1 | | 126.1 | | + | 1 | 6 | -13 | | -22 | -25 |
|  |  | | 90.1 | | + | 1 | 6 | -13 | | -38 | -18 |
| Thiamethoxam | 292.0 | | 211.1 | | + | 1 | 5 | -14 | | -13 | -23 |
|  |  | | 181.0 | | + | 1 | 5 | -14 | | -24 | -19 |
| Thiamethoxam-d3 | 296.6 | | 214.1 | | + | 1 | 5 | -11 | | -12 | -23 |
|  |  | | 184.1 | | + | 1 | 5 | -14 | | -24 | -19 |
| Thiazopyr | 397.0 | | 377.1 | | + | 1 | 10 | -14 | | -23 | -18 |
| Timolol | 317.1 | | 261.1 | | + | 1 | 12 | -16 | | -17 | -30 |
| Tramadol | 264.1 | | 58.2 | | + | 1 | 14 | -19 | | -16 | -22 |
| Tramadol-13C1, d3 | 268.3 | | 58.2 | | + | 1 | 14 | -13 | | -23 | -24 |
| Trimethoprim | 291.1 | | 230.1 | | + | 1 | 5 | -30 | | -25 | -26 |
|  |  | | 123.2 | | + | 1 | 5 | -15 | | -28 | -25 |
| Trimethoprim-d3 | 294.1 | | 230.2 | | + | 1 | 5 | -20 | | -25 | -25 |
|  |  | | 123.2 | | + | 1 | 5 | -11 | | -26 | -12 |
| Valsartan | 436.4 | | 291.2 | | + | 1 | 5 | -15 | | -18 | -14 |
|  |  | | 235.2 | | + | 1 | 5 | -12 | | -17 | -16 |
| Venlafaxine | 278.2 | | 58.1 | | + | 1 | 6 | -13 | | -19 | -22 |
|  |  | | 260.2 | | + | 1 | 6 | -13 | | -15 | -20 |
| Venlafaxine-d6 | 284.2 | | 64.2 | | + | 1 | 6 | -20 | | -23 | -26 |
|  |  | | 266.3 | | + | 1 | 6 | -30 | | -13 | -27 |
| Verapamil | 455.2 | | 165.2 | | + | 1 | 4 | -30 | | -29 | -30 |
|  |  | | 414.4 | | + | 1 | 4 | -16 | | -16 | -15 |
| Verapamil-d3 | 458.2 | | 165.2 | | + | 1 | 4 | -30 | | -29 | -29 |
|  |  | | 306.3 | | + | 1 | 4 | -30 | | -26 | -15 |
| Warfarin | 309.2 | | 163.1 | | + | 1 | 5 | -15 | | -16 | -19 |
|  |  | | 251.1 | | + | 1 | 5 | -15 | | -20 | -29 |
| Ziprasidone | 413.1 | | 194.1 | | + | 1 | 4 | -20 | | -30 | -20 |
|  |  | | 159.1 | | + | 1 | 4 | -20 | | -40 | -20 |

**Table S3.** Method repeatability for sediment extraction and analysis at two concentrations (n=5).

|  | **Repeatability (n= 5)** | |
| --- | --- | --- |
| **Compound** | **20 ng∙g^-1^** | **100 ng∙g^-1^** |
| Amitriptyline | 9 | 13 |
| Azoxystrobin | 6 | 13 |
| Benzatropine | 15 | 11 |
| Benzoylecgonine | 8 | 5 |
| Bezafibrate | 6 | 5 |
| Bisoprolol | 18 | 10 |
| Busipirone | 12 | 3 |
| Carbamazepine | 14 | 12 |
| CBZ-10,11-epoxide | 4 | 7 |
| Chloramphenicol | 14 | 5 |
| Citalopram | 9 | 10 |
| Diazepam | 8 | 2 |
| Diruon | 6 | 2 |
| Haloperidol | 14 | 11 |
| Ketamine | 7 | 4 |
| Lidocaine | 6 | 3 |
| MDMA | 16 | 7 |
| Metoprolol | 3 | 3 |
| Nordiazepam | 10 | 5 |
| Orphenadrine | 20 | 9 |
| Oxazepam | 15 | 15 |
| Propamocarb | 9 | 11 |
| Propranolol | 9 | 10 |
| Sertraline | 18 | 5 |
| Sulfamethazine | 5 | 1 |
| Sulfapyridine | 4 | 2 |
| Tamsulosin | 10 | 7 |
| Thiacloprid | 5 | 3 |
| Tramadol | 2 | 1 |
| Trimethoprim | 3 | 3 |
| Venlafaxine | 17 | 17 |
| Verapamil | 2 | 1 |

**Table S4:** SIL-IS correction applied for the specified analytes that were detected in the present study. SIL-IS were not applied to analytes that demonstrated acceptable linearity (R^2^>0.98)

| **Compound** | **SIL-IS** |
| --- | --- |
| Acetamiprid | - |
| Alprazolam | - |
| Amitriptyline | Amitriptyline-d3 |
| Atrazine | - |
| Azithromycin | Clarithromycin-d3 |
| Azoxystrobin | Nordiazepam-d5 |
| Benzatropine | - |
| Benzoylecgonine | Benzoylecgonine, BZE-d3 |
| Betaxolol | Betaxolol-d7 |
| Bezafibrate | - |
| Bisoprolol | - |
| Buspirone | - |
| Carbamazepine | - |
| CBZ.epoxide | - |
| Chloramphenicol | - |
| Citalopram | Sertraline-d3 |
| Clarithromycin | Clarithromycin-d3 |
| Clofibric.acid | - |
| Clozapine | Amitriptyline-d3 |
| Cocaine | Cocaine-d3 |
| Diazepam | Diazepam-d6 |
| Diclofenac | - |
| Diphenhydramine | Lorazepam-d4 |
| Diuron | Lorazepam-d4 |
| Fenuron | - |
| Fluoxetine | Fluoxetine-d6 |
| Haloperidol | Haloperidol-d4 |
| Hydrochlorothiazide | - |
| Imidacloprid | - |
| Indomethacin | Tramadol-13C1, d3 |
| Ketamine | Ketamine-d4 |
| Lidocaine | Cocaine-d3 |
| MDMA | MDMA-d5 |
| Memantine | - |
| Methamphetamine | Amphetamine-d6 |
| Methylphenidate | Methylphenidate-d9 |
| Metoprolol | Metoprolol-d7 |
| Nicotine | Nicotine-d4 |
| Nordiazepam | Nordiazepam-d5 |
| Nortriptyline | Nortriptyline-d3 |
| Orphenadrine | - |
| Oxazepam | Oxazepam-d5 |
| Propamocarb | - |
| Propanolol | - |
| Pymetrozine | - |
| Risperidone | Risperidone-d4 |
| Rizatriptan | - |
| Ronidazole | - |
| Salbutamol | - |
| Sertraline | Sertraline-d3 |
| Simazine | - |
| Sulfadimethoxine | Sulfamethazine-d4 |
| Sulfamethazine | Sulfamethazine-d4 |
| Sulfamonomethoxine | Sulfamethazine-d4 |
| Sulfapyridine | Sulfamethazine-d4 |
| Sulfisoxazole | Sulfamethazine-d4 |
| Tacrine | - |
| Tamsulosin | - |
| Temazepam | Temazepam-d5 |
| Terbutryn | - |
| Thiacloprid | - |
| Thiamethoxam | Thiamethoxam-d3 |
| Timolol | - |
| Tramadol | Tramadol-13C1, d3 |
| Trimethoprim | Trimethoprim-d3 |
| Venlafaxine | Venlafaxine-d6 |
| Verapamil | Verapamil-d3 |
| Warfarin | - |


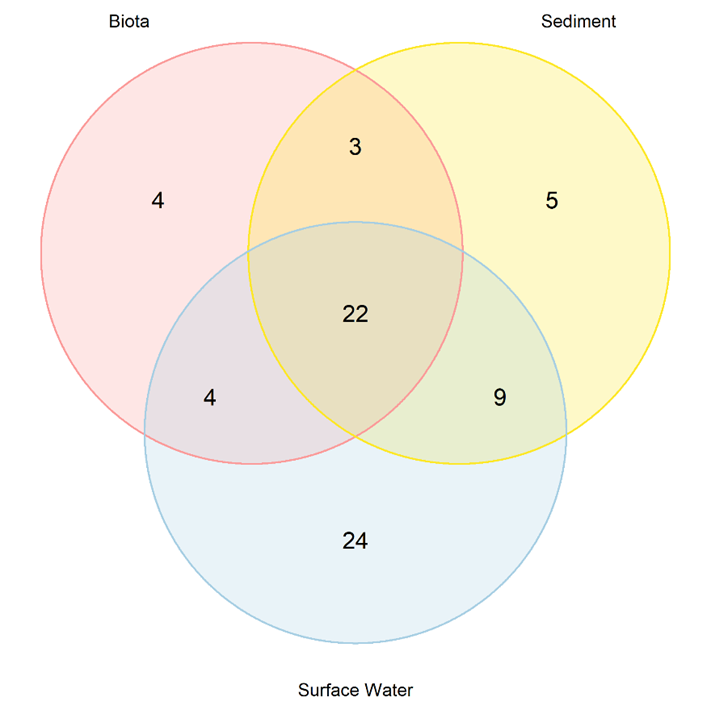
**S3.0** Comparison of compartmental contamination

**Figure S1.** Venn diagram showing a comparison of the number of chemicals detected across the three compartments analysed in the Colne estuary including sediment, surface water and biota. Biota includes all macroinvertebrate species sampled.
